# Supplementary figures and images for: Formalin-free tissue embedding is less hazardous and results in better DNA quality
Source: PLoS One. 2024 Dec 30;19(12):e0316107. doi: 10.1371/journal.pone.0316107 (PMC11684726; doi:10.1371/journal.pone.0316107)

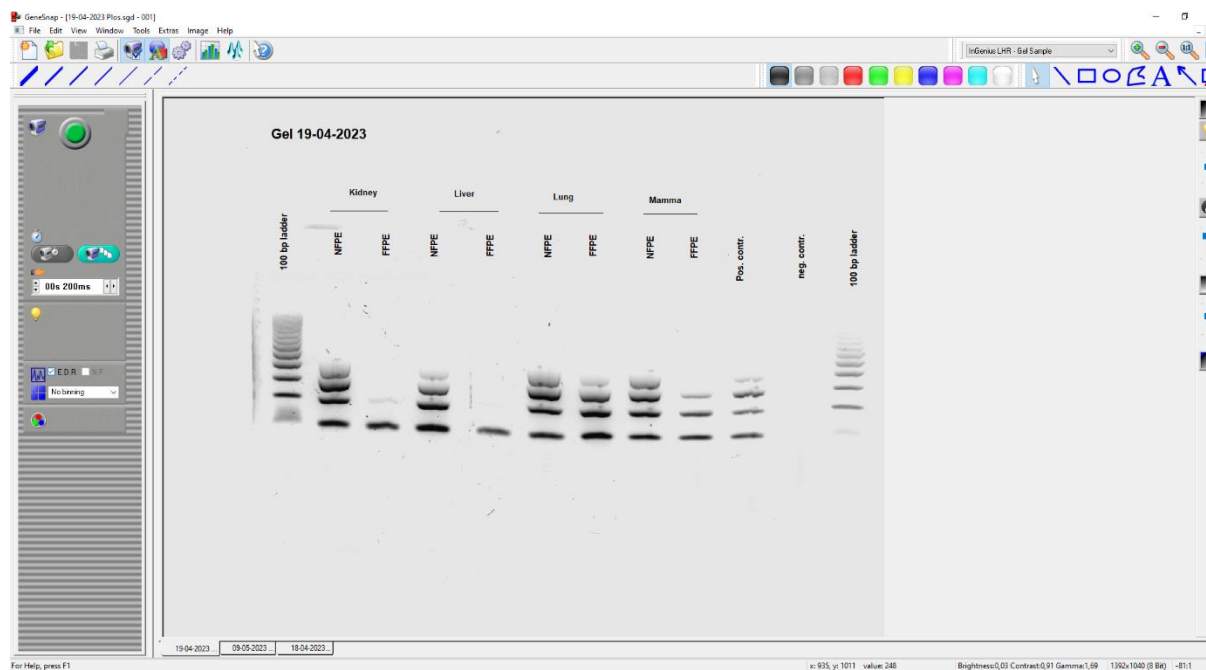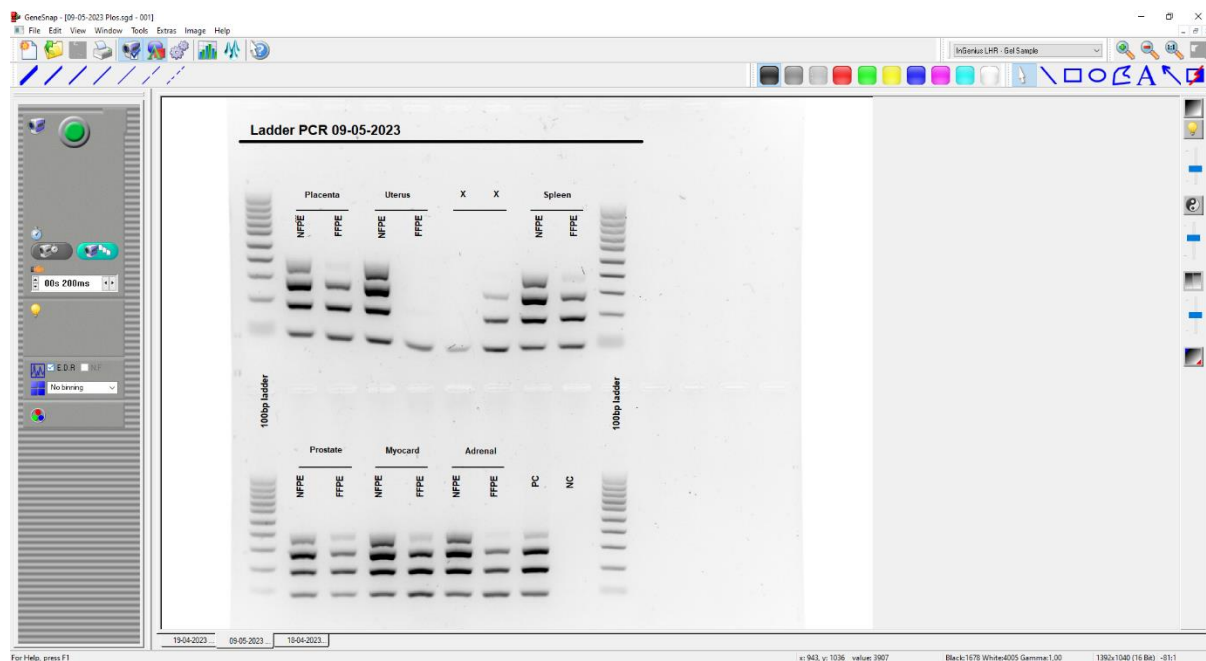

Supplement: S1 Raw images — (PDF) [file pone.0316107.s001.pdf]
