# Supplementary material for: Formalin-free tissue embedding is less hazardous and results in better DNA quality
Source: PLoS One. 2024 Dec 30;19(12):e0316107. doi: 10.1371/journal.pone.0316107 (PMC11684726; doi:10.1371/journal.pone.0316107)
Supplement: S1 Data — (PDF) [file pone.0316107.s002.pdf]

Supplementary data read length histograms

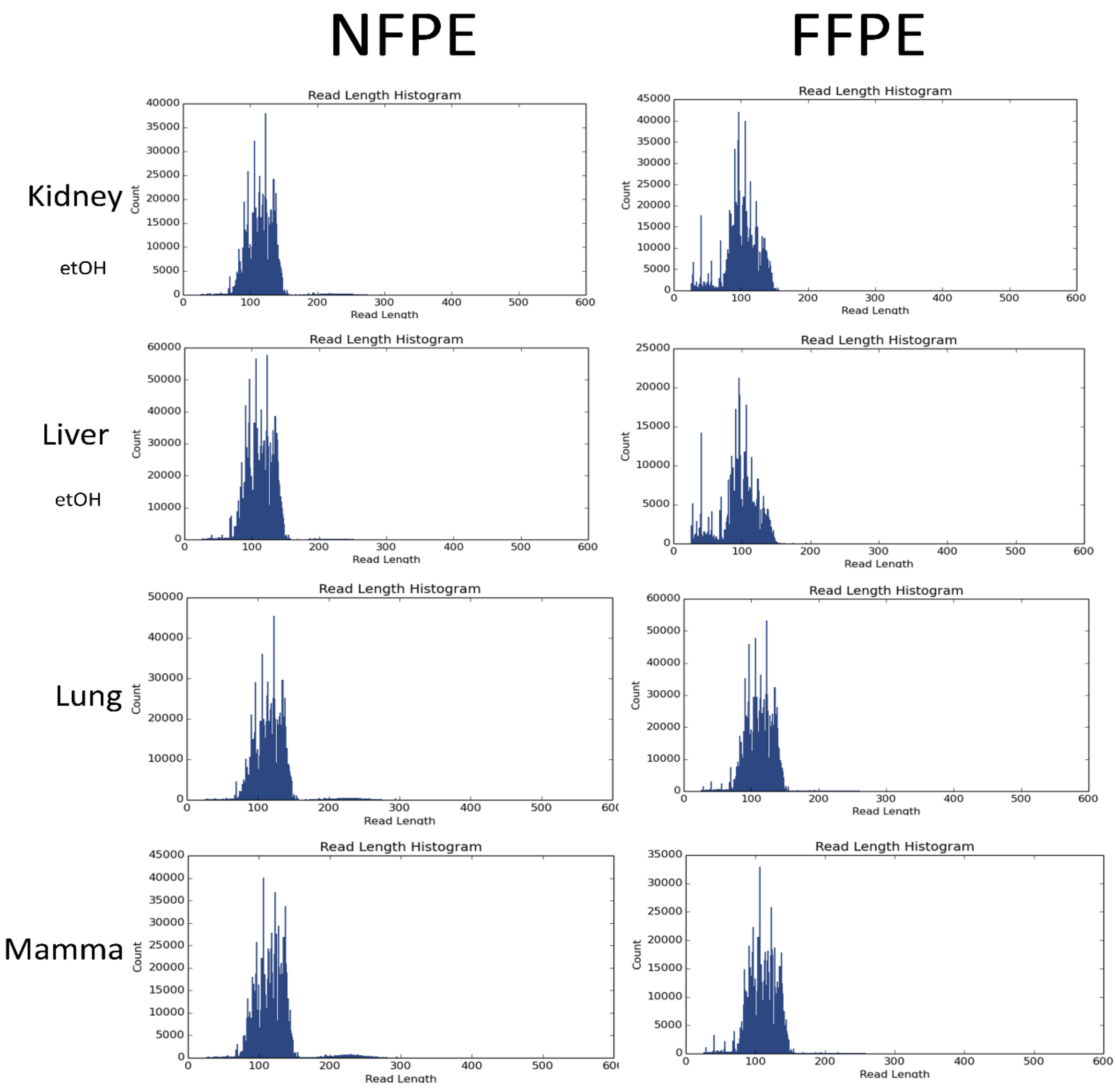

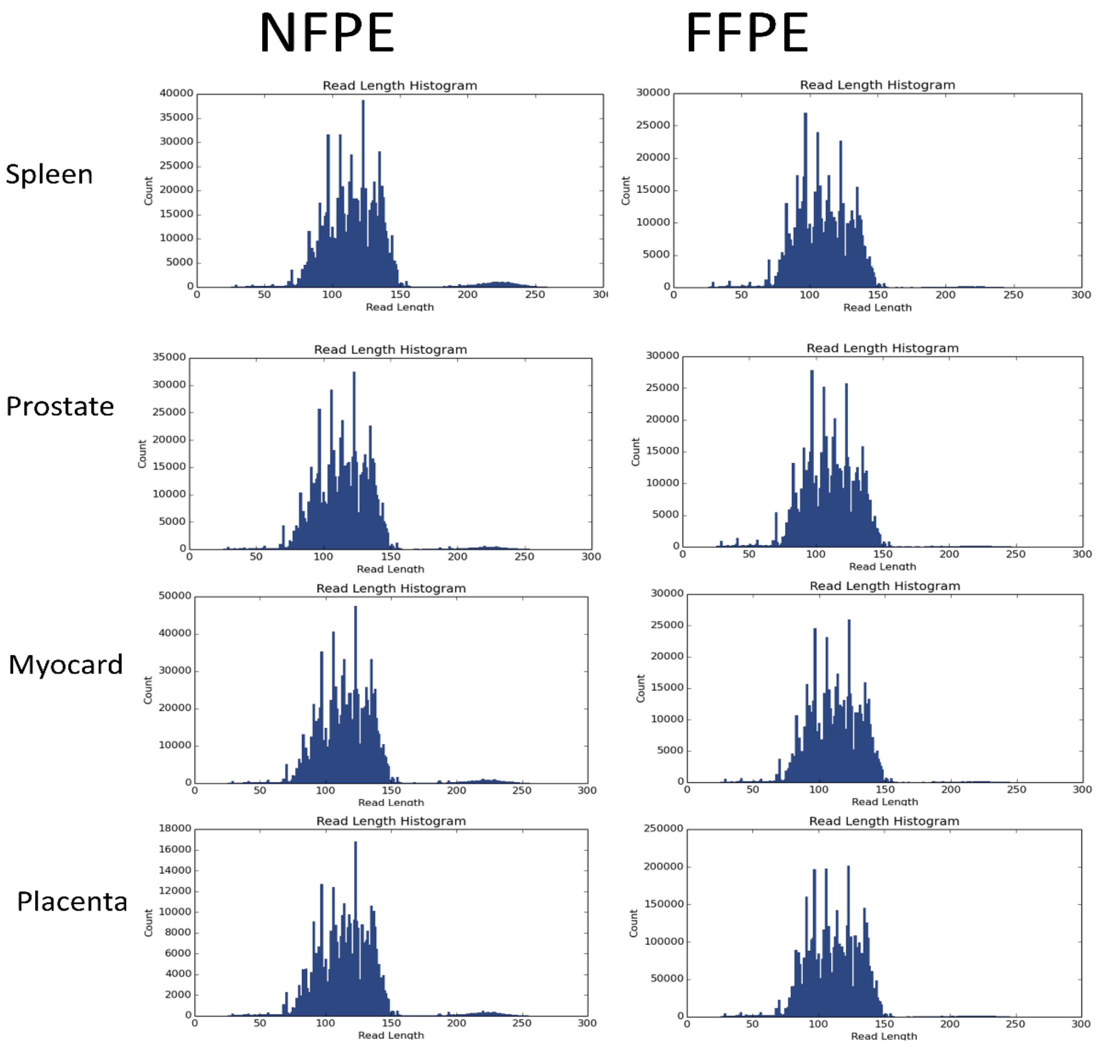
NFPE
FFPE
Spleen
Prostate
Myocard
Placenta
Read Length Histogram
Count
Read Length

Supplementary data Fixation artefacts

% VAF too high
Artefact (C>T/G>A in 1-10% VAF range)
No artefact (non-C>T/G>A in 1-10% VAF range)
% VAF too low

NFPE Mamma

chr5:176523597	G/G	A	FGFR4	p.?	100
chr5:176523562	A/A	C	FGFR4	p.?	100
chr2:29416572	C/C	T	ALK	p.Ile1461Val	100
chr4:55141055	G/G	A	PDGFRA	p.Pro567=	99,95
chr5:176517326	C/C	T	FGFR4	p.?	99,9
chr4:55566266	A/A	G	KIT	p.?	99,89
chr4:1807894	A/A	G	FGFR3	p.Thr651=	99,83
chr12:25386063	A/A	C	KRAS	p.?	99,7
chr5:176517797	T/T	C	FGFR4	p.Pro136Leu	99,55
chr4:55097835	G/C	G	PDGFRA	p.?	62,36
chr3:178922274	C/A	C	PIK3CA	p.?	54,24
chr7:92286918	A/G	A	CDK6	p.?	52,78
chr7:128845511	G/A	G	SMO	p.Val270Ile	50,38
chr4:55593464	A/C	A	KIT	p.Met541Leu	50,08
chr1:65310489	T/C	T	JAK1	p.Pro733=	50,03
chr12:58144665	C/T	C	CDK4	p.?	49,97
chr12:56477694	A/T	A	ERBB3	p.?	49,87
chr4:55133726	T/G	T	PDGFRA	p.Gly313=	49,42
chr10:43613843	G/T	G	RET	p.Leu769=	48,95
chr10:43615633	C/G	C	RET	p.Ser904=	48,8
chr4:1797741	T/C	T	FGFR3	p.?	42,14
chr12:25364863	C/T	C	KRAS	p.?	39,74
chr5:112043384	T/G	T	APC		35,81
chr5:176517985	A/G	A	FGFR4	p.Ala161=	18,79
chr4:55599321	A/A	A	KIT		0,4
chr10:43613839	A/A	A	RET		0,35
chr3:178916945	A/A	A	PIK3CA		0,35
chr15:66727441	T/T	T	MAP2K1		0,33
chr3:178936083	A/A	A	PIK3CA		0,3
chr12:58145431	G/G	G	CDK4		0,3
chr12:58145430	C/C	C	CDK4		0,3
chr3:178936095	A/A	A	PIK3CA		0,3
chr1:65310517	C/C	C	JAK1		0,28
chr7:116423474	T/T	T	MET		0,25
chr4:55594258	T/T	T	KIT		0,25
chrX:66943543	C/C	C	AR		0,25
chr19:3115013	G/G	G	GNA11		0,25
chr19:3115012	C/C	C	GNA11		0,25
chr12:58145436	T/T	T	CDK4		0,25
chr2:29445270	A/A	A	ALK		0,25
chr10:123274794	T/T	T	FGFR2		0,22
chr4:55593661	T/T	T	KIT		0,2
chr11:533874	T/T	T	HRAS		0,2
chr1:115256529	T/T	T	NRAS		0,2
chr4:55599260	A/A	A	KIT		0,2
chr3:41266100	T/T	T	CTNNB1		0,2
chr12:56481659	G/G	G	ERBB3		0,2
chr7:116417464	A/A	A	MET		0,2
chr7:55221821	G/G	G	EGFR		0,2
chr3:178938860	A/A	A	PIK3CA		0,2
chr3:178916924	C/C	C	PIK3CA		0,2
chrX:66943585	A/A	A	AR		0,2
chr17:37868208	C/C	C	ERBB2		0,2
chr12:56482537	G/G	G	ERBB3		0,2
chr12:25380276	T/T	T	KRAS		0,2
chr7:55249070	A/A	A	EGFR ...(2)		0,2
chr7:55241708	G/G	G	EGFR		0,2
chr7:55241678	A/A	A	EGFR		0,2

chr4:55599348	T/T	T	KIT	0,2
chr4:1806152	G/G	G	FGFR3	0,2
chr3:178952088	A/A	A	PIK3CA	0,2
chr3:178952065	G/G	G	PIK3CA	0,2
chr3:178947827	G/G	G	PIK3CA	0,2
chr3:178936098	A/A	A	PIK3CA	0,2
chr3:178936071	A/A	A	PIK3CA	0,2
chr3:178927980	T/T	T	PIK3CA	0,2
chr3:178916729	A/A	A	PIK3CA	0,2
chr3:41266112	T/T	T	CTNNB1	0,2
chr2:29445213	A/A	A	ALK	0,2
chr1:11184574	A/A	A	MTOR	0,2
chr4:55599320	G/G	G	KIT	0,15
chr3:178936082	G/G	G	PIK3CA	0,15
chr1:11188078	C/C	C	MTOR	0,15
chr9:5073770	G/G	G	JAK2	0,15
chr19:4117551	A/A	A	MAP2K2	0,15
chr3:178952072	A/A	A	PIK3CA	0,15
chr17:37880220	T/T	T	ERBB2	0,15
chrX:66943552	A/A	A	AR	0,15
chr19:3118942	A/A	A	GNA11	0,15
chr15:90631839	T/T	T	IDH2	0,15
chr15:66774131	G/G	G	MAP2K1	0,15
chr10:123247516	T/T	T	FGFR2	0,15
chr10:43617415	A/A	A	RET	0,15
chr10:43609948	T/T	T	RET	0,15
chr10:43609102	T/T	T	RET	0,15
chr9:80412493	C/C	C	GNAQ	0,15
chr7:140453134	T/T	T	BRAF	0,15
chr7:116423414	A/A	A	MET	0,15
chr6:152419926	A/A	A	ESR1	0,15
chr6:117638347	C/C	C	ROS1	0,15
chr4:55599339	A/A	A	KIT	0,15
chr4:1803565	G/G	G	FGFR3	0,15
chr3:178952082	C/C	C	PIK3CA	0,15
chr3:178952079	A/A	A	PIK3CA	0,15
chr3:178952063	A/A	A	PIK3CA	0,15
chr3:178952007	A/A	A	PIK3CA	0,15
chr3:178947826	T/T	T	PIK3CA	0,15
chr3:178938935	A/A	A	PIK3CA	0,15
chr3:178921552	A/A	A	PIK3CA	0,15
chr3:178916725	C/C	C	PIK3CA	0,15

FFPE Mamma

chr5:176523597	G/G	A	FGFR4	p.?	100
chr5:176523562	A/A	C	FGFR4	p.?	100
chr4:55566266	A/A	G	KIT	p.?	99,91
chr4:55141055	G/G	A	PDGFRA	p.Pro567=	99,85
chr12:25386063	A/A	C	KRAS	p.?	99,85
chr4:1807894	A/A	G	FGFR3	p.Thr651=	99,82
chr2:29416572	C/C	T	ALK	p.Ile1461Val	99,65
chr5:176517797	T/T	C	FGFR4	p.Pro136Leu	99,54
chr5:176517326	C/C	T	FGFR4	p.?	99,3
chr4:55097835	G/C	G	PDGFRA	p.?	68,6
chr7:128845511	G/A	G	SMO	p.Val270Ile	55,99
chr4:55133726	T/G	T	PDGFRA	p.Gly313=	53,02
chr12:58144665	C/T	C	CDK4	p.?	52,57
chr10:43615633	C/G	C	RET	p.Ser904=	52,12
chr10:43613843	G/T	G	RET	p.Leu769=	52,1
chr4:55593464	A/C	A	KIT	p.Met541Leu	49,98
chr7:92286918	A/G	A	CDK6	p.?	49,87
chr12:56477694	A/T	A	ERBB3	p.?	48,85
chr1:65310489	T/C	T	JAK1	p.Pro733=	48,78
chr3:178922274	C/A	C	PIK3CA	p.?	43,94
chr4:1797741	T/C	T	FGFR3	p.?	40,67
chr5:176517985	A/G	A	FGFR4	p.Ala161=	38,83
chr5:112043384	T/G	T	APC		37,94
chr12:25364863	C/T	C	KRAS	p.?	34,87
chr12:58145430	C/C	C	CDK4		0,96
chr1:11190804	C/C	C	MTOR		0,92
chr15:90631934	C/C	C	IDH2		0,82
chr3:178916924	C/C	C	PIK3CA		0,75
chr7:140481403	C/C	C	BRAF		0,7
chr17:37880261	G/G	G	ERBB2		0,7
chr3:178936070	G/G	G	PIK3CA		0,7
chr3:41266097	G/G	G	CTNNB1		0,7
chr7:128846398	C/C	C	SMO		0,65
chr3:178928079	G/G	G	PIK3CA		0,65
chr3:178916936	G/G	G	PIK3CA		0,65
chr9:5073770	G/G	G	JAK2		0,6
chr17:37868208	C/C	C	ERBB2		0,6
chr3:178916876	G/G	G	PIK3CA		0,6
chr3:178916945	A/A	A	PIK3CA		0,55
chr12:56482537	G/G	G	ERBB3		0,55
chr6:117638347	C/C	C	ROS1		0,55
chr3:178952065	G/G	G	PIK3CA		0,5
chr1:115252203	G/G	G	NRAS		0,5
chr3:178916728	G/G	G	PIK3CA		0,48
chr1:162724598	C/C	C	DDR2		0,46
chr12:25398282	C/C	C	KRAS		0,45
chr7:55211080	G/G	G	EGFR		0,45
chr17:37881332	G/G	G	ERBB2		0,45
chr7:55241708	G/G	G	EGFR		0,45
chr3:178952091	G/G	G	PIK3CA		0,45
chr3:178952078	G/G	G	PIK3CA		0,45
chr3:178921548	G/G	G	PIK3CA		0,45
chr3:178916726	G/G	G	PIK3CA		0,41
chr12:56481659	G/G	G	ERBB3		0,4
chr4:55599261	G/G	G	KIT		0,4
chr3:178952074	G/G	G	PIK3CA		0,4
chr2:29445213	A/A	A	ALK		0,4
chr15:90631839	T/T	T	IDH2		0,4

chr7:116417464	A/A	A	MET		0,37
chr4:1807889	A/A	A	FGFR3		0,36
chr19:4117551	A/A	A	MAP2K2		0,35
chr17:37868207	T/T	T	ERBB2		0,35
chr12:56482341	G/G	G	ERBB3		0,35
chr4:55599332	G/G	G	KIT		0,35
chr3:178952090	G/G	G	PIK3CA		0,35
chr3:178936095	A/A	A	PIK3CA		0,35
chr1:115252204	C/C	C	NRAS		0,35
chr1:11217230	C/C	C	MTOR		0,35
chr10:123274794	T/T	T	FGFR2		0,34
chr7:140481402	C/C	C	BRAF		0,3
chr7:140453134	T/T	T	BRAF		0,3
chrX:66943585	A/A	A	AR		0,3
chr12:25380276	T/T	T	KRAS		0,3
chr3:178952084	C/C	C	PIK3CA		0,3
chr3:178952064	T/T	T	PIK3CA		0,3
chr2:29445270	A/A	A	ALK		0,3
chr2:29432664	C/C	C	ALK		0,3
chr1:115258748	C/C	C	CSDE1 ...(	p.?	0,3
chr15:66727441	T/T	T	MAP2K1		0,29
chr12:58145436	T/T	T	CDK4		0,27
chr12:25378562	C/C	C	KRAS		0,26
chr1:115258747	C/C	C	CSDE1 ...(	p.?	0,25
chr3:41266107	T/T	T	CTNNB1		0,25
chr19:17945969	C/C	C	JAK3		0,25
chr17:37881000	G/G	G	ERBB2		0,25
chr12:56478817	G/G	G	ERBB3		0,25
chr12:25398284	C/C	C	KRAS		0,25
chr11:534286	C/C	C	HRAS		0,25
chr4:55593660	C/C	C	KIT		0,25
chr4:55593610	T/T	T	KIT		0,25
chr3:178952073	T/T	T	PIK3CA		0,25
chr3:178952072	A/A	A	PIK3CA		0,25
chr3:178952049	C/C	C	PIK3CA		0,25
chr3:178938934	G/G	G	PIK3CA		0,25
chr3:178936073	C/C	C	PIK3CA		0,25
chr3:41266104	G/G	G	CTNNB1		0,25
chr2:212488718	G/G	G	ERBB4		0,25
chr1:65311204	T/T	T	JAK1		0,25
chr7:116417463	C/C	C	MET		0,22
chr7:55249071	C/C	C	EGFR ...(2)		0,22

NFPE Placenta

chr10:43613843	T/T	G	RET	p.Leu769=	100
chr5:176523562	A/A	C	FGFR4	p.?	100
chr4:55529199	TA/TA	T	KIT	p.?	100
chr2:29416366	C/C	G	ALK	p.Asp1529Glu	99,93
chr4:1807894	A/A	G	FGFR3	p.Thr651=	99,89
chr12:25400206	T/T	G	KRAS	p.?	99,77
chr4:55141055	G/G	A	PDGFRA	p.Pro567=	99,77
chr2:29416572	C/C	T	ALK	p.Ile1461Val	99,75
chr5:176517797	T/T	C	FGFR4	p.Pro136Leu	99,68
chr5:176517326	C/C	T	FGFR4	p.?	99,68
chr5:176523597	G/G	A	FGFR4	p.?	99,62
chr7:55249063	A/A	G	EGFR ...(2	p.Gln787=	99,33
chr7:55198980	CT/.	C	EGFR	p.?	68,56
chr12:25389220	A/G	A	KRAS	p.?	56,93
chr12:25389182	A/G	A	KRAS	p.?	56,75
chr10:43615633	C/G	C	RET	p.Ser904=	52,81
chr12:25386940	C/T	C	KRAS	p.?	52,3
chr12:25386063	C/A	C	KRAS	p.?	51,81
chr12:25391239	G/C	G	KRAS	p.?	51,76
chr8:128750540	A/G	A	MYC	p.Asn26Ser	51,16
chr2:29445458	G/T	G	ALK	p.Gly1125=	50,67
chr2:29416615	G/A	G	ALK	p.Thr1446=	49,75
chr1:65310489	T/C	T	JAK1	p.Pro733=	49,56
chr3:178922274	C/A	C	PIK3CA	p.?	48,02
chr4:55566266	G/A	G	KIT	p.?	47,03
chr7:92286918	A/G	A	CDK6	p.?	46,83
chr2:209113192	G/A	G	IDH1	p.Gly105=	46,34
chr4:1797741	T/C	T	FGFR3	p.?	45,24
chr5:176517985	A/G	A	FGFR4	p.Ala161=	41,94
chr3:182672831	CTTTT	CTT	DCUN1D1	p.?	21,24
chr7:55198979	CTTTT	TCT	EGFR	p.?	14,63
chr3:178952065	G/G	G	PIK3CA		0,66
chr3:41266112	T/T	T	CTNNB1		0,54
chr3:178916945	A/A	A	PIK3CA		0,5
chr3:178936091	G/G	G	PIK3CA		0,45
chr10:123279674	G/G	G	FGFR2		0,45
chr10:43609949	G/G	G	RET		0,45
chr1:115258745	C/C	C	CSDE1 ...(	p.?	0,44
chr4:55593609	G/G	G	KIT		0,41
chr7:116411990	C/C	C	MET		0,39
chr15:90631838	C/C	C	IDH2		0,37
chr4:55594258	T/T	T	KIT		0,36
chr19:17945969	C/C	C	JAK3		0,35
chr10:43617398	G/G	G	RET		0,35
chr2:209113113	G/G	G	IDH1		0,35
chr1:65311204	T/T	T	JAK1		0,35
chr7:55249004	A/A	A	EGFR ...(2)		0,34
chr4:1807889	A/A	A	FGFR3		0,33
chr3:178952087	C/C	C	PIK3CA		0,33
chr3:178952018	A/A	A	PIK3CA		0,33
chr7:55211079	A/A	A	EGFR		0,33
chr17:37881332	G/G	G	ERBB2		0,33
chr7:128845572	G/G	G	SMO		0,32
chr7:128845571	C/C	C	SMO		0,32
chr4:1803565	G/G	G	FGFR3		0,31
chr15:90631839	T/T	T	IDH2		0,31
chr4:55599321	A/A	A	KIT		0,31
chr2:29445213	A/A	A	ALK		0,3

chr2:29432664	C/C	C	ALK	0,3
chr12:56482341	G/G	G	ERBB3	0,3
chr10:43613839	A/A	A	RET	0,3
chr7:116423473	A/A	A	MET	0,3
chr3:178936073	C/C	C	PIK3CA	0,3
chr7:140453133	T/T	T	BRAF	0,3
chr1:11188078	C/C	C	MTOR	0,29
chr12:25378561	G/G	G	KRAS	0,29
chr10:43609102	T/T	T	RET	0,28
chr7:55211080	G/G	G	EGFR	0,27
chr3:178916729	A/A	A	PIK3CA	0,27
chr12:56478854	G/G	G	ERBB3	0,26
chr3:178922324	G/G	G	PIK3CA	0,26
chr15:66774131	G/G	G	MAP2K1	0,26
chr4:55599333	A/A	A	KIT	0,25
chr4:55599332	G/G	G	KIT	0,25
chr3:178936071	A/A	A	PIK3CA	0,25
chrX:66943585	A/A	A	AR	0,25
chr10:43617415	A/A	A	RET	0,25
chr3:178947827	G/G	G	PIK3CA	0,25
chr3:178921548	G/G	G	PIK3CA	0,25
chr15:90631935	G/G	G	IDH2	0,25
chr1:115256536	C/C	C	NRAS	0,25
chr15:66727441	T/T	T	MAP2K1	0,24
chr7:140453136	A/A	A	BRAF	0,24
chr3:41266136	T/T	T	CTNNB1	0,24
chr7:55221821	G/G	G	EGFR	0,24
chr3:41266107	T/T	T	CTNNB1	0,23
chr2:29443695	G/G	G	ALK	0,23
chr3:41266104	G/G	G	CTNNB1	0,23
chr3:41266109	C/C	C	CTNNB1	0,23
chr3:41266098	A/A	A	CTNNB1	0,23
chr4:55141036	T/T	T	PDGFRA	0,23
chr10:43609096	T/T	T	RET	0,22
chr19:3115013	G/G	G	GNA11	0,21
chr7:55241708	G/G	G	EGFR	0,21
chr12:25398285	C/C	C	KRAS	0,21
chr3:178916725	C/C	C	PIK3CA	0,2
chr4:55599347	G/G	G	KIT	0,2
chr9:80412493	C/C	C	GNAQ	0,2
chr17:37881617	G/G	G	ERBB2	0,2
chr12:56481659	G/G	G	ERBB3	0,2

FFPE placenta

chr10:43613843	T/T	G	RET	p.Leu769=	100
chr4:55529199	TA/TA	T	KIT	p.?	100
chr4:1807894	A/A	G	FGFR3	p.Thr651=	99,9
chr2:29416572	C/C	T	ALK	p.Ile1461Val	99,9
chr12:25400206	T/T	G	KRAS	p.?	99,9
chr5:176523562	A/A	C	FGFR4	p.?	99,9
chr2:29416366	C/C	G	ALK	p.Asp1529Glu	99,9
chr4:55141055	G/G	A	PDGFRA	p.Pro567=	99,7
chr5:176523597	G/G	A	FGFR4	p.?	99,65
chr5:176517326	C/C	T	FGFR4	p.?	99,45
chr5:176517797	T/T	C	FGFR4	p.Pro136Leu	99,15
chr7:55249063	A/A	G	EGFR ...(2	p.Gln787=	98,85
chr12:25386063	C/A	C	KRAS	p.?	54,97
chr12:25391239	G/C	G	KRAS	p.?	54,84
chr3:178922274	C/A	C	PIK3CA	p.?	54,76
chr12:25389182	A/G	A	KRAS	p.?	54,2
chr12:25389220	A/G	A	KRAS	p.?	53,73
chr2:29416615	G/A	G	ALK	p.Thr1446=	52,88
chr7:92286918	A/G	A	CDK6	p.?	52,55
chr1:65310489	T/C	T	JAK1	p.Pro733=	52,33
chr4:55566266	G/A	G	KIT	p.?	51,98
chr10:43615633	C/G	C	RET	p.Ser904=	51,73
chr2:29445458	G/T	G	ALK	p.Gly1125=	49,55
chr2:209113192	G/A	G	IDH1	p.Gly105=	48,87
chr8:128750540	A/G	A	MYC	p.Asn26Ser	47,99
chr12:25386940	C/T	C	KRAS	p.?	46,21
chr4:1797741	T/C	T	FGFR3	p.?	43,02
chr5:176517985	A/G	A	FGFR4	p.Ala161=	9,95
chr3:178916891	G/G	G	PIK3CA		0,8
chr3:178916876	G/G	G	PIK3CA		0,8
chr3:178936097	G/G	G	PIK3CA		0,75
chr1:115258748	C/C	C	CSDE1 ...(	p.?	0,7
chr7:55211080	G/G	G	EGFR		0,65
chr19:3115012	C/C	C	GNA11		0,6
chr1:115258747	C/C	C	CSDE1 ...(	p.?	0,55
chr19:3115013	G/G	G	GNA11		0,55
chr3:178952091	G/G	G	PIK3CA		0,55
chr7:128845571	C/C	C	SMO		0,5
chr7:140481411	C/C	C	BRAF		0,5
chr3:178936091	G/G	G	PIK3CA		0,5
chr3:178916854	G/G	G	PIK3CA		0,5
chr15:90631934	C/C	C	IDH2		0,45
chr17:37881616	C/C	C	ERBB2		0,45
chr7:140481412	C/C	C	BRAF		0,45
chr4:1806120	G/G	G	FGFR3		0,4
chrX:66943543	C/C	C	AR		0,4
chr17:37881332	G/G	G	ERBB2		0,4
chr12:56482537	G/G	G	ERBB3		0,4
chr3:178921548	G/G	G	PIK3CA		0,4
chr1:115256536	C/C	C	NRAS		0,4
chr19:3118942	A/A	A	GNA11		0,35
chr15:66774131	G/G	G	MAP2K1		0,35
chr11:534288	C/C	C	HRAS		0,35
chr4:1803565	G/G	G	FGFR3		0,35
chr3:178952090	G/G	G	PIK3CA		0,35
chr3:178947827	G/G	G	PIK3CA		0,35
chr1:115258745	C/C	C	CSDE1 ...(	p.?	0,35
chr3:41266100	T/T	T	CTNNB1		0,3

chr12:25378561	G/G	G	KRAS		0,3
chr17:37879658	G/G	G	ERBB2		0,3
chr17:37868208	C/C	C	ERBB2		0,3
chr15:66727441	T/T	T	MAP2K1		0,3
chr12:58145436	T/T	T	CDK4		0,3
chr12:25380283	C/C	C	KRAS		0,3
chr4:1806119	G/G	G	FGFR3		0,3
chr3:178952065	G/G	G	PIK3CA		0,3
chr3:178928079	G/G	G	PIK3CA		0,3
chr3:41266104	G/G	G	CTNNB1		0,3
chr3:12645699	G/G	G	RAF1		0,3
chr2:212488718	G/G	G	ERBB4		0,3
chr2:29432664	C/C	C	ALK		0,3
chr1:162724598	C/C	C	DDR2		0,3
chr1:115252203	G/G	G	NRAS		0,3
chr3:41266136	T/T	T	CTNNB1		0,25
chr3:41266137	C/C	C	CTNNB1		0,25
chr1:115256529	T/T	T	NRAS		0,25
chr7:55241677	G/G	G	EGFR		0,25
chr4:1806152	G/G	G	FGFR3		0,25
chr3:178936082	G/G	G	PIK3CA		0,25
chr12:25398281	C/C	C	KRAS		0,25
chr7:55221822	C/C	C	EGFR		0,25
chr4:1803564	C/C	C	FGFR3		0,25
chr15:90631935	G/G	G	IDH2		0,25
chr14:105246551	C/C	C	AKT1		0,25
chr10:123274773	C/C	C	FGFR2		0,25
chr10:43617397	C/C	C	RET		0,25
chr7:140481403	C/C	C	BRAF		0,25
chr6:152419926	A/A	A	ESR1		0,25
chr3:41266113	C/C	C	CTNNB1		0,25
chr2:29443613	C/C	C	ALK		0,25
chr1:11217231	A/A	A	MTOR		0,25
chr4:55593609	G/G	G	KIT		0,21
chr17:37880997	G/G	G	ERBB2		0,2
chr7:55249005	G/G	G	EGFR ...(2)		0,2
chr4:55599320	G/G	G	KIT		0,2
chr7:116412044	G/G	G	MET	p.?	0,2
chrX:66943549	T/T	T	AR		0,2
chr12:25398282	C/C	C	KRAS		0,2
chr7:140453155	C/C	C	BRAF		0,2
chr3:178936073	C/C	C	PIK3CA		0,2

NFPE Adrenal

chr10:43613843	T/T	G	RET	p.Leu769=	100
chr7:92286918	G/G	A	CDK6	p.?	100
chr5:176523597	G/G	A	FGFR4	p.?	100
chr3:178922274	A/A	C	PIK3CA	p.?	99,95
chr12:25391239	C/C	G	KRAS	p.?	99,95
chr5:176523562	A/A	C	FGFR4	p.?	99,89
chr5:176517326	C/C	T	FGFR4	p.?	99,85
chr2:29416572	C/C	T	ALK	p.Ile1461Val	99,85
chr4:55566266	A/A	G	KIT	p.?	99,84
chr4:1807894	A/A	G	FGFR3	p.Thr651=	99,75
chr7:55249063	A/A	G	EGFR ...(2	p.Gln787=	99,7
chr4:55141055	G/G	A	PDGFRA	p.Pro567=	99,7
chr12:58144665	T/T	C	CDK4	p.?	99,68
chr12:25400206	T/T	G	KRAS	p.?	99,64
chr5:176517797	T/T	C	FGFR4	p.Pro136Leu	99,59
chr12:56477694	A/T	A	ERBB3	p.?	51,13
chr4:1807922	G/A	G	FGFR3	p.?	50,38
chr4:55152040	C/T	C	PDGFRA	p.Val824=	50,3
chr1:65310489	T/C	T	JAK1	p.Pro733=	50,24
chr5:176517985	A/G	A	FGFR4	p.Ala161=	32,2
chr12:25398281	C/C	C	KRAS		0,65
chr7:140481403	C/C	C	BRAF		0,55
chr3:178952048	G/G	G	PIK3CA		0,55
chr3:178916726	G/G	G	PIK3CA		0,48
chr7:55249071	C/C	C	EGFR ...(2)		0,45
chr7:55211080	G/G	G	EGFR		0,45
chr7:140453155	C/C	C	BRAF		0,4
chr4:55599333	A/A	A	KIT		0,4
chr3:178938935	A/A	A	PIK3CA		0,35
chr12:56482341	G/G	G	ERBB3		0,35
chr11:534286	C/C	C	HRAS		0,35
chr10:43609102	T/T	T	RET		0,35
chr4:55599332	G/G	G	KIT		0,35
chr4:55593660	C/C	C	KIT		0,35
chr10:123274773	C/C	C	FGFR2		0,34
chr15:66727441	T/T	T	MAP2K1		0,32
chr7:140481402	C/C	C	BRAF		0,3
chr3:178916890	C/C	C	PIK3CA		0,3
chr12:56482537	G/G	G	ERBB3		0,3
chr12:25398285	C/C	C	KRAS		0,3
chr12:25378561	G/G	G	KRAS		0,3
chr10:123279674	G/G	G	FGFR2		0,3
chr10:43609948	T/T	T	RET		0,3
chr7:55241707	G/G	G	EGFR		0,3
chr4:1803564	C/C	C	FGFR3		0,3
chr3:178952085	A/A	A	PIK3CA		0,3
chr3:178952082	C/C	C	PIK3CA		0,3
chr3:178936070	G/G	G	PIK3CA		0,3
chr3:41266136	T/T	T	CTNNB1		0,25
chr2:209113112	C/C	C	IDH1		0,25
chr1:115256529	T/T	T	NRAS		0,25
chr4:1806119	G/G	G	FGFR3		0,25
chr7:128845572	G/G	G	SMO		0,25
chr3:178936082	G/G	G	PIK3CA		0,25
chr1:115258744	C/C	C	CSDE1 ...(	p.?	0,25
chr17:37881332	G/G	G	ERBB2		0,25
chr15:90631839	T/T	T	IDH2		0,25
chr7:140453137	C/C	C	BRAF		0,25

chr3:178952078	G/G	G	PIK3CA	0,25
chr3:178928079	G/G	G	PIK3CA	0,25
chr3:178916876	G/G	G	PIK3CA	0,25
chr3:41266104	G/G	G	CTNNB1	0,25
chr3:41266097	G/G	G	CTNNB1	0,25
chr2:209113113	G/G	G	IDH1	0,25
chr2:29445213	A/A	A	ALK	0,25
chr3:178916729	A/A	A	PIK3CA	0,21
chr3:178916725	C/C	C	PIK3CA	0,21
chr3:178916728	G/G	G	PIK3CA	0,21
chr1:65310517	C/C	C	JAK1	0,21
chr3:178916945	A/A	A	PIK3CA	0,2
chr3:178916936	G/G	G	PIK3CA	0,2
chr7:140453133	T/T	T	BRAF	0,2
chr12:25398282	C/C	C	KRAS	0,2
chr4:55599260	A/A	A	KIT	0,2
chr1:115256536	C/C	C	NRAS	0,2
chr1:11190804	C/C	C	MTOR	0,2
chr19:4117551	A/A	A	MAP2K2	0,2
chr7:55221821	G/G	G	EGFR	0,2
chr7:55211079	A/A	A	EGFR	0,2
chr3:178936097	G/G	G	PIK3CA	0,2
chr17:37868207	T/T	T	ERBB2	0,2
chr15:90631935	G/G	G	IDH2	0,2
chr15:66729162	C/C	C	MAP2K1	0,2
chr10:43617398	G/G	G	RET	0,2
chr10:43617397	C/C	C	RET	0,2
chr7:55241678	A/A	A	EGFR	0,2
chr3:178952091	G/G	G	PIK3CA	0,2
chr3:178952090	G/G	G	PIK3CA	0,2
chr3:178952088	A/A	A	PIK3CA	0,2
chr3:178952065	G/G	G	PIK3CA	0,2
chr3:178952018	A/A	A	PIK3CA	0,2
chr3:178938860	A/A	A	PIK3CA	0,2
chr3:178936092	A/A	A	PIK3CA	0,2
chr3:178936091	G/G	G	PIK3CA	0,2
chr3:178936073	C/C	C	PIK3CA	0,2
chr3:178921549	T/T	T	PIK3CA	0,2
chr1:115252204	C/C	C	NRAS	0,2
chr12:58145436	T/T	T	CDK4	0,16
chr12:58145431	G/G	G	CDK4	0,16
chr4:55593603	T/T	T	KIT	0,15

FFPE Adrenal

chr12:25400206	T/T	G	KRAS	p.?	100
chr10:43613843	T/T	G	RET	p.Leu769=	100
chr5:176523597	G/G	A	FGFR4	p.?	100
chr5:176523562	A/A	C	FGFR4	p.?	100
chr5:176517326	C/C	T	FGFR4	p.?	100
chr12:25391239	C/C	G	KRAS	p.?	99,93
chr4:55141055	G/G	A	PDGFRA	p.Pro567=	99,9
chr3:178922274	A/A	C	PIK3CA	p.?	99,88
chr7:55249063	A/A	G	EGFR ...(2	p.Gln787=	99,83
chr4:1807894	A/A	G	FGFR3	p.Thr651=	99,75
chr2:29416572	C/C	T	ALK	p.Ile1461Val	99,75
chr5:176517797	T/T	C	FGFR4	p.Pro136Leu	99,74
chr12:58144665	T/T	C	CDK4	p.?	99,69
chr4:55566266	A/A	G	KIT	p.?	99,54
chr7:92286918	G/G	A	CDK6	p.?	98,84
chr12:56477694	A/T	A	ERBB3	p.?	57,99
chr4:55152040	C/T	C	PDGFRA	p.Val824=	53,4
chr4:1807922	G/A	G	FGFR3	p.?	53,14
chr1:65310489	T/C	T	JAK1	p.Pro733=	45,45
chr5:176517985	A/G	A	FGFR4	p.Ala161=	29,7
chr15:90631935	G/G	G	IDH2		1,87
chr3:178916891	G/G	G	PIK3CA		1,31
chr3:178952078	G/G	G	PIK3CA		1,24
chr17:37880261	G/G	G	ERBB2		1,02
chr12:56478817	G/G	G	ERBB3		1
chr7:116412044	G/G	G	MET	p.?	0,92
chr3:178922324	G/G	G	PIK3CA		0,9
chr3:178936070	G/G	G	PIK3CA		0,87
chr3:178936073	C/C	C	PIK3CA		0,87
chr3:178938934	G/G	G	PIK3CA		0,85
chr12:56478854	G/G	G	ERBB3		0,85
chr17:37881617	G/G	G	ERBB2		0,83
chr15:66729162	C/C	C	MAP2K1		0,8
chr14:105246551	C/C	C	AKT1		0,77
chr3:178916854	G/G	G	PIK3CA		0,76
chr3:178952074	G/G	G	PIK3CA		0,75
chr1:115256536	C/C	C	NRAS		0,75
chr3:178916936	G/G	G	PIK3CA		0,7
chr3:178952090	G/G	G	PIK3CA		0,7
chr3:178952084	C/C	C	PIK3CA		0,7
chr12:25380283	C/C	C	KRAS		0,7
chr1:11190804	C/C	C	MTOR		0,68
chr3:41266137	C/C	C	CTNNB1		0,66
chr17:37881000	G/G	G	ERBB2		0,65
chr4:1803564	C/C	C	FGFR3		0,65
chr1:115252204	C/C	C	NRAS		0,63
chr1:162724598	C/C	C	DDR2		0,6
chr4:1806152	G/G	G	FGFR3		0,58
chr17:37880997	G/G	G	ERBB2		0,56
chr7:55241677	G/G	G	EGFR		0,55
chr11:534289	C/C	C	HRAS		0,55
chr7:140481411	C/C	C	BRAF		0,55
chr3:178952048	G/G	G	PIK3CA		0,54
chr4:55599261	G/G	G	KIT		0,53
chr1:115258748	C/C	C	CSDE1 ...(	p.?	0,5
chr1:115258745	C/C	C	CSDE1 ...(	p.?	0,5
chr3:178922364	G/G	G	PIK3CA		0,48
chr10:123274773	C/C	C	FGFR2		0,47

chr3:41266136	T/T	T	CTNNB1		0,46
chr12:25398284	C/C	C	KRAS		0,45
chr12:25398282	C/C	C	KRAS		0,45
chr17:37879903	C/C	C	ERBB2		0,45
chr10:123247516	T/T	T	FGFR2		0,45
chr3:41266097	G/G	G	CTNNB1		0,45
chr2:212488718	G/G	G	ERBB4		0,45
chr4:55599347	G/G	G	KIT		0,45
chr4:55599333	A/A	A	KIT		0,45
chr2:29443613	C/C	C	ALK		0,43
chr3:178952091	G/G	G	PIK3CA		0,43
chr1:65310518	G/G	G	JAK1		0,42
chr4:1806119	G/G	G	FGFR3		0,4
chr4:1803565	G/G	G	FGFR3		0,4
chr2:29445258	C/C	C	ALK		0,4
chr1:65310517	C/C	C	JAK1		0,35
chr3:178916945	A/A	A	PIK3CA		0,35
chr1:115256529	T/T	T	NRAS		0,35
chr17:37879658	G/G	G	ERBB2		0,35
chr2:29445270	A/A	A	ALK		0,35
chr11:534285	C/C	C	HRAS		0,35
chr9:80412493	C/C	C	GNAQ		0,35
chr7:55211080	G/G	G	EGFR		0,35
chr3:178947827	G/G	G	PIK3CA		0,35
chr3:41266125	C/C	C	CTNNB1		0,35
chr3:41266101	C/C	C	CTNNB1		0,35
chr3:178916725	C/C	C	PIK3CA		0,33
chr3:178952081	G/G	G	PIK3CA		0,32
chr7:116412045	T/T	T	MET	p.?	0,31
chr1:115258744	C/C	C	CSDE1 ...(	p.?	0,3
chr19:17945969	C/C	C	JAK3		0,3
chrX:66943585	A/A	A	AR		0,3
chr12:25378562	C/C	C	KRAS		0,3
chr10:43609097	G/G	G	RET		0,3
chr7:128846398	C/C	C	SMO		0,3
chr3:178936071	A/A	A	PIK3CA		0,29
chr3:178936082	G/G	G	PIK3CA		0,29
chr15:90631839	T/T	T	IDH2		0,29
chr7:55242511	G/G	G	EGFR		0,28
chr7:55249071	C/C	C	EGFR ...(2)		0,28
chr3:178916876	G/G	G	PIK3CA		0,28
chr3:178952076	A/A	A	PIK3CA		0,27

NFPE lung

chr4:55566266	A/A	G	KIT	p.?	99,95
chr5:176523597	G/G	A	FGFR4	p.?	99,93
chr2:29416572	C/C	T	ALK	p.Ile1461Va	99,9
chr12:25386063	A/A	C	KRAS	p.?	99,9
chr5:176523562	A/A	C	FGFR4	p.?	99,86
chr5:176517326	C/C	T	FGFR4	p.?	99,85
chr4:55141055	G/G	A	PDGFRA	p.Pro567=	99,85
chr4:1807894	A/A	G	FGFR3	p.Thr651=	99,8
chr5:176517797	T/T	C	FGFR4	p.Pro136Le	99,4
chr7:55249063	G/A	G	EGFR ...(2)	p.Gln787=	53,25
chr12:25386940	C/T	C	KRAS	p.?	52,53
chr10:43613843	G/T	G	RET	p.Leu769=	52,33
chr12:25400206	G/T	G	KRAS	p.?	52,09
chr4:55529199	T/TA	T	KIT	p.?	50
chr12:25389182	A/G	A	KRAS	p.?	49,62
chr5:112043384	T/G	T	APC		49,32
chr3:178922274	C/A	C	PIK3CA	p.?	48,4
chr5:176517985	A/G	A	FGFR4	p.Ala161=	21,9
chr3:178916945	A/A	A	PIK3CA		0,5
chr19:4117551	A/A	A	MAP2K2		0,4
chr3:178952090	G/G	G	PIK3CA		0,4
chr3:178928079	G/G	G	PIK3CA		0,4
chr10:123274794	T/T	T	FGFR2		0,35
chr3:41266125	C/C	C	CTNNB1		0,35
chr4:55593609	G/G	G	KIT		0,3
chr17:37879658	G/G	G	ERBB2		0,3
chr7:55259502	A/A	A	EGFR		0,3
chr7:55221822	C/C	C	EGFR		0,3
chr4:55599321	A/A	A	KIT		0,3
chr3:178952019	C/C	C	PIK3CA		0,3
chr1:65311204	T/T	T	JAK1		0,3
chr4:55593603	T/T	T	KIT		0,25
chr3:41266136	T/T	T	CTNNB1		0,25
chr12:58145436	T/T	T	CDK4		0,25
chr3:41266107	T/T	T	CTNNB1		0,25
chr17:37881332	G/G	G	ERBB2		0,25
chr12:56478854	G/G	G	ERBB3		0,25
chr7:140481402	C/C	C	BRAF		0,25
chr3:178938860	A/A	A	PIK3CA		0,25
chr3:178936092	A/A	A	PIK3CA		0,25
chr3:41266106	A/A	A	CTNNB1		0,25
chr2:29445270	A/A	A	ALK		0,25
chr1:11217230	C/C	C	MTOR		0,25
chr3:178936082	G/G	G	PIK3CA		0,2
chr7:55249004	A/A	A	EGFR ...(2)		0,2
chr6:152419926	A/A	A	ESR1		0,2
chr7:128845571	C/C	C	SMO		0,2
chr3:178938935	A/A	A	PIK3CA		0,2
chr3:178922325	A/A	A	PIK3CA		0,2
chr1:115252203	G/G	G	NRAS		0,2
chr17:37868207	T/T	T	ERBB2		0,2
chr12:25398285	C/C	C	KRAS		0,2
chr7:116423474	T/T	T	MET		0,2
chr7:55249071	C/C	C	EGFR ...(2)		0,2
chr7:55241708	G/G	G	EGFR		0,2
chr4:55594221	A/A	A	KIT		0,2
chr3:178952088	A/A	A	PIK3CA		0,2
chr3:41266104	G/G	G	CTNNB1		0,2

chr3:41266098	A/A	A	CTNNB1		0,2
chr2:29443613	C/C	C	ALK		0,2
chr17:37880997	G/G	G	ERBB2		0,15
chr4:55593613	T/T	T	KIT		0,15
chr19:17949164	T/T	T	JAK3		0,15
chr12:25398282	C/C	C	KRAS		0,15
chr7:140453145	A/A	A	BRAF		0,15
chr7:140453155	C/C	C	BRAF		0,15
chr7:116412045	T/T	T	MET	p.?	0,15
chr19:17948862	A/A	A	JAK3		0,15
chr15:66727441	T/T	T	MAP2K1		0,15
chr12:56478817	G/G	G	ERBB3		0,15
chr7:55249070	A/A	A	EGFR ...(2)		0,15
chr3:178952072	A/A	A	PIK3CA		0,15
chr19:3118942	A/A	A	GNA11		0,15
chr19:3115013	G/G	G	GNA11		0,15
chr17:37880220	T/T	T	ERBB2		0,15
chr17:37879903	C/C	C	ERBB2		0,15
chr15:90631839	T/T	T	IDH2		0,15
chr15:90631838	C/C	C	IDH2		0,15
chr12:58145431	G/G	G	CDK4		0,15
chr12:25380283	C/C	C	KRAS		0,15
chr12:25380276	T/T	T	KRAS		0,15
chr10:123274795	A/A	A	FGFR2		0,15
chr10:123247516	T/T	T	FGFR2		0,15
chr10:43609097	G/G	G	RET		0,15
chr9:80412493	C/C	C	GNAQ		0,15
chr7:140453134	T/T	T	BRAF		0,15
chr7:116423475	G/G	G	MET		0,15
chr7:116423473	A/A	A	MET		0,15
chr7:116423428	T/T	T	MET		0,15
chr7:55221821	G/G	G	EGFR		0,15
chr7:55211079	A/A	A	EGFR		0,15
chr4:55599333	A/A	A	KIT		0,15
chr4:55594258	T/T	T	KIT		0,15
chr4:1806119	G/G	G	FGFR3		0,15
chr3:178952081	G/G	G	PIK3CA		0,15
chr3:178952076	A/A	A	PIK3CA		0,15
chr3:178936097	G/G	G	PIK3CA		0,15
chr3:178936095	A/A	A	PIK3CA		0,15
chr3:178936083	A/A	A	PIK3CA		0,15
chr3:178936070	G/G	G	PIK3CA		0,15

FFPE lung

chr2:29416572	C/C	T	ALK	p.Ile1461V	100
chr5:176523562	A/A	C	FGFR4	p.?	99,93
chr4:55141055	G/G	A	PDGFRA	p.Pro567=	99,9
chr4:1807894	A/A	G	FGFR3	p.Thr651=	99,9
chr4:55566266	A/A	G	KIT	p.?	99,8
chr5:176523597	G/G	A	FGFR4	p.?	99,79
chr12:25386063	A/A	C	KRAS	p.?	99,65
chr5:176517797	T/T	C	FGFR4	p.Pro136Le	99,6
chr5:176517326	C/C	T	FGFR4	p.?	99,55
chr12:25400206	G/T	G	KRAS	p.?	54,79
chr3:178922274	C/A	C	PIK3CA	p.?	51,78
chr4:55529199	T/TA	T	KIT	p.?	50,76
chr12:25386940	C/T	C	KRAS	p.?	49,85
chr10:43613843	G/T	G	RET	p.Leu769=	49,15
chr12:25389182	A/G	A	KRAS	p.?	48,75
chr7:55249063	G/A	G	EGFR ...(2	p.Gln787=	45,85
chr5:112043384	T/G	T	APC		45,55
chr5:176517985	A/G	A	FGFR4	p.Ala161=	40,73
chr7:140481412	C/C	C	BRAF		0,7
chr7:128845572	G/G	G	SMO		0,65
chr3:178916876	G/G	G	PIK3CA		0,65
chr12:25398282	C/C	C	KRAS		0,6
chr17:37880261	G/G	G	ERBB2		0,6
chr15:90631935	G/G	G	IDH2		0,6
chr12:56478854	G/G	G	ERBB3		0,55
chr1:65310518	G/G	G	JAK1		0,5
chr7:128846398	C/C	C	SMO		0,5
chr3:178952091	G/G	G	PIK3CA		0,5
chr7:55241707	G/G	G	EGFR		0,5
chr12:25398284	C/C	C	KRAS		0,45
chr3:178952081	G/G	G	PIK3CA		0,45
chr12:56482537	G/G	G	ERBB3		0,45
chr12:25398285	C/C	C	KRAS		0,45
chr4:55599261	G/G	G	KIT		0,45
chr3:178936097	G/G	G	PIK3CA		0,45
chr3:41266125	C/C	C	CTNNB1		0,45
chr3:12645699	G/G	G	RAF1		0,45
chr3:41266136	T/T	T	CTNNB1		0,41
chr7:140481402	C/C	C	BRAF		0,4
chr7:55221821	G/G	G	EGFR		0,4
chr1:65310517	C/C	C	JAK1		0,4
chr10:123279674	G/G	G	FGFR2		0,4
chr10:43617398	G/G	G	RET		0,4
chr4:55599347	G/G	G	KIT		0,4
chr2:212488718	G/G	G	ERBB4		0,4
chr1:115258745	C/C	C	CSDE1 ...(	p.?	0,4
chr7:55241677	G/G	G	EGFR		0,35
chr17:37868208	C/C	C	ERBB2		0,35
chr15:90631934	C/C	C	IDH2		0,35
chr7:55221822	C/C	C	EGFR		0,35
chr17:37879903	C/C	C	ERBB2		0,35
chr12:25380276	T/T	T	KRAS		0,35
chr7:55241708	G/G	G	EGFR		0,35
chr3:178922324	G/G	G	PIK3CA		0,35
chr3:178916726	G/G	G	PIK3CA		0,35
chr19:17949164	T/T	T	JAK3		0,3
chr4:1803564	C/C	C	FGFR3		0,3
chr2:29445270	A/A	A	ALK		0,3

chr17:37879658	G/G	G	ERBB2	0,3
chr10:43617415	A/A	A	RET	0,3
chr7:116423475	G/G	G	MET	0,3
chr7:116411990	C/C	C	MET	0,3
chr4:55599332	G/G	G	KIT	0,3
chr4:1806153	C/C	C	FGFR3	0,3
chr4:1806120	G/G	G	FGFR3	0,3
chr3:178952084	C/C	C	PIK3CA	0,3
chr3:178916891	G/G	G	PIK3CA	0,3
chr3:41266110	A/A	A	CTNNB1	0,3
chr1:11190804	C/C	C	MTOR	0,3
chr1:11189847	A/A	A	MTOR	0,3
chr1:11188078	C/C	C	MTOR	0,3
chr7:55249004	A/A	A	EGFR ...(2)	0,25
chr3:178938935	A/A	A	PIK3CA	0,25
chr1:65311204	T/T	T	JAK1	0,25
chr17:37881332	G/G	G	ERBB2	0,25
chr11:534285	C/C	C	HRAS	0,25
chr10:43617416	T/T	T	RET	0,25
chr9:80412493	C/C	C	GNAQ	0,25
chr3:178952063	A/A	A	PIK3CA	0,25
chr3:178936070	G/G	G	PIK3CA	0,25
chr3:178928079	G/G	G	PIK3CA	0,25
chr3:178922325	A/A	A	PIK3CA	0,25
chr3:178916924	C/C	C	PIK3CA	0,25
chr3:178916729	A/A	A	PIK3CA	0,25
chr3:41266104	G/G	G	CTNNB1	0,25
chr1:11217231	A/A	A	MTOR	0,25
chr1:11217230	C/C	C	MTOR	0,25
chr3:41266137	C/C	C	CTNNB1	0,2
chr4:55593661	T/T	T	KIT	0,2
chr17:37880997	G/G	G	ERBB2	0,2
chr4:55593613	T/T	T	KIT	0,2
chr2:209113112	C/C	C	IDH1	0,2
chr12:56481660	C/C	C	ERBB3	0,2
chr7:140481403	C/C	C	BRAF	0,2
chr1:11184574	A/A	A	MTOR	0,2
chr19:17945969	C/C	C	JAK3	0,2
chr10:43613839	A/A	A	RET	0,2
chr10:43609096	T/T	T	RET	0,2
chr7:55249071	C/C	C	EGFR ...(2)	0,2
chr7:55211079	A/A	A	EGFR	0,2

NFPE Myocard

chr5:176523597	G/G	A	FGFR4	p.?	100
chr5:176523562	A/A	C	FGFR4	p.?	100
chr4:55141055	G/G	A	PDGFRA	p.Pro567=	99,85
chr5:176517326	C/C	T	FGFR4	p.?	99,85
chr2:29416572	C/C	T	ALK	p.Ile1461V	99,85
chr4:1807894	A/A	G	FGFR3	p.Thr651=	99,85
chr4:55566266	A/A	G	KIT	p.?	99,75
chr5:176517797	T/T	C	FGFR4	p.Pro136Le	99,6
chr12:25386063	A/A	C	KRAS	p.?	99,5
chr12:25389182	A/G	A	KRAS	p.?	55,5
chr5:112043384	T/G	T	APC		52,2
chr10:43613843	G/T	G	RET	p.Leu769=	50,75
chr4:55529199	T/TA	T	KIT	p.?	50,58
chr3:178922274	C/A	C	PIK3CA	p.?	50,38
chr7:55249063	G/A	G	EGFR ...(2	p.Gln787=	49,98
chr12:25400206	G/T	G	KRAS	p.?	47,56
chr12:25386940	C/T	C	KRAS	p.?	46,74
chr5:176517985	A/G	A	FGFR4	p.Ala161=	41,78
chr19:17949164	T/T	T	JAK3		0,55
chr10:123279674	G/G	G	FGFR2		0,55
chr2:29432664	C/C	C	ALK		0,5
chr4:1807889	A/A	A	FGFR3		0,45
chr3:178952018	A/A	A	PIK3CA		0,45
chr3:178916891	G/G	G	PIK3CA		0,45
chr7:128845572	G/G	G	SMO		0,4
chr15:90631935	G/G	G	IDH2		0,4
chr4:55599321	A/A	A	KIT		0,4
chr3:178952091	G/G	G	PIK3CA		0,4
chr3:178952081	G/G	G	PIK3CA		0,4
chr4:55599260	A/A	A	KIT		0,35
chr1:115252203	G/G	G	NRAS		0,35
chr7:55241678	A/A	A	EGFR		0,35
chr12:25398282	C/C	C	KRAS		0,35
chr9:80412493	C/C	C	GNAQ		0,35
chr4:55594258	T/T	T	KIT		0,35
chr3:178952082	C/C	C	PIK3CA		0,35
chr3:178952048	G/G	G	PIK3CA		0,35
chr1:115256529	T/T	T	NRAS		0,3
chrX:66943549	T/T	T	AR		0,3
chr7:140481412	C/C	C	BRAF		0,3
chr7:55211079	A/A	A	EGFR		0,3
chr17:37881000	G/G	G	ERBB2		0,3
chr15:66729163	C/C	C	MAP2K1		0,3
chr7:55211080	G/G	G	EGFR		0,3
chr4:55599347	G/G	G	KIT		0,3
chr4:55599261	G/G	G	KIT		0,3
chr3:178952049	C/C	C	PIK3CA		0,3
chr3:178928080	A/A	A	PIK3CA		0,3
chr3:178928079	G/G	G	PIK3CA		0,3
chr3:178927980	T/T	T	PIK3CA		0,3
chr3:178916936	G/G	G	PIK3CA		0,3
chr1:115258745	C/C	C	CSDE1 ...(	p.?	0,3
chr2:209113112	C/C	C	IDH1		0,25
chr17:37880261	G/G	G	ERBB2		0,25
chr17:37880220	T/T	T	ERBB2		0,25
chr17:37879658	G/G	G	ERBB2		0,25
chr12:25398285	C/C	C	KRAS		0,25
chr10:123274795	A/A	A	FGFR2		0,25

chr7:116423473	A/A	A	MET		0,25
chr3:178936097	G/G	G	PIK3CA		0,25
chr3:178916726	G/G	G	PIK3CA		0,25
chr3:41266112	T/T	T	CTNNB1		0,25
chr1:11189847	A/A	A	MTOR		0,25
chr19:3118942	A/A	A	GNA11		0,2
chr12:25398284	C/C	C	KRAS		0,2
chr7:140453133	T/T	T	BRAF		0,2
chr17:37868208	C/C	C	ERBB2		0,2
chr3:178936082	G/G	G	PIK3CA		0,2
chr10:43613839	A/A	A	RET		0,2
chr7:140453155	C/C	C	BRAF		0,2
chr7:128845571	C/C	C	SMO		0,2
chr7:116412044	G/G	G	MET	p.?	0,2
chr7:55241708	G/G	G	EGFR		0,2
chr7:55241707	G/G	G	EGFR		0,2
chr3:178952019	C/C	C	PIK3CA		0,2
chr1:11217231	A/A	A	MTOR		0,2
chr19:3115012	C/C	C	GNA11		0,2
chr15:90631934	C/C	C	IDH2		0,2
chr12:25380276	T/T	T	KRAS		0,2
chr11:534286	C/C	C	HRAS		0,2
chr10:123279675	G/G	G	FGFR2		0,2
chr6:152419926	A/A	A	ESR1		0,2
chr4:55593610	T/T	T	KIT		0,2
chr3:178952072	A/A	A	PIK3CA		0,2
chr3:178952065	G/G	G	PIK3CA		0,2
chr3:178952007	A/A	A	PIK3CA		0,2
chr3:178938860	A/A	A	PIK3CA		0,2
chr3:178916945	A/A	A	PIK3CA		0,2
chr2:209113113	G/G	G	IDH1		0,2
chr2:29445258	C/C	C	ALK		0,2
chr2:29443696	A/A	A	ALK		0,2
chr1:162724598	C/C	C	DDR2		0,2
chr15:66727441	T/T	T	MAP2K1		0,17
chr4:55593661	T/T	T	KIT		0,15
chr4:55593609	G/G	G	KIT		0,15
chr9:5073770	G/G	G	JAK2		0,15
chr7:55249004	A/A	A	EGFR ...(2)		0,15
chrX:66943552	A/A	A	AR		0,15
chr12:25378649	T/T	T	KRAS		0,15
chr4:55594221	A/A	A	KIT		0,15

FFPE Myocard

chr5:176523562	A/A	C	FGFR4	p.?	100
chr4:55141055	G/G	A	PDGFRA	p.Pro567=	99,9
chr4:1807894	A/A	G	FGFR3	p.Thr651=	99,86
chr5:176523597	G/G	A	FGFR4	p.?	99,85
chr2:29416572	C/C	T	ALK	p.Ile1461Va	99,8
chr12:25386063	A/A	C	KRAS	p.?	99,75
chr5:176517326	C/C	T	FGFR4	p.?	99,68
chr4:55566266	A/A	G	KIT	p.?	99,65
chr5:176517797	T/T	C	FGFR4	p.Pro136Le	99,47
chr12:25386940	C/T	C	KRAS	p.?	51,94
chr7:55249063	G/A	G	EGFR ...(2	p.Gln787=	51,93
chr4:55529199	T/TA	T	KIT	p.?	51,3
chr12:25389182	A/G	A	KRAS	p.?	50,5
chr3:178922274	C/A	C	PIK3CA	p.?	49,22
chr12:25400206	G/T	G	KRAS	p.?	49,19
chr10:43613843	G/T	G	RET	p.Leu769=	48,9
chr5:112043384	T/G	T	APC		46,92
chr5:176517985	A/G	A	FGFR4	p.Ala161=	33,98
chr2:212488718	G/G	G	ERBB4		0,7
chr12:56481660	C/C	C	ERBB3		0,65
chr3:41266103	G/G	G	CTNNB1		0,6
chr15:66729163	C/C	C	MAP2K1		0,6
chr17:37880997	G/G	G	ERBB2		0,59
chr3:178952091	G/G	G	PIK3CA		0,55
chr4:55593660	C/C	C	KIT		0,5
chr3:178952064	T/T	T	PIK3CA		0,5
chr3:178916725	C/C	C	PIK3CA		0,5
chr2:209113112	C/C	C	IDH1		0,5
chr4:55599332	G/G	G	KIT		0,45
chr3:178952074	G/G	G	PIK3CA		0,45
chr3:178938934	G/G	G	PIK3CA		0,45
chr4:1806153	C/C	C	FGFR3		0,42
chr4:1806120	G/G	G	FGFR3		0,42
chr4:55593609	G/G	G	KIT		0,41
chr1:65310518	G/G	G	JAK1		0,41
chr9:5073770	G/G	G	JAK2		0,4
chr12:56478817	G/G	G	ERBB3		0,4
chr7:116423475	G/G	G	MET		0,4
chr3:178936097	G/G	G	PIK3CA		0,4
chr2:29443613	C/C	C	ALK		0,4
chr1:115252203	G/G	G	NRAS		0,4
chr11:534289	C/C	C	HRAS		0,39
chr11:534288	C/C	C	HRAS		0,39
chr7:55241677	G/G	G	EGFR		0,35
chr17:37881332	G/G	G	ERBB2		0,35
chr15:66729162	C/C	C	MAP2K1		0,35
chr7:55211079	A/A	A	EGFR		0,35
chr3:178916945	A/A	A	PIK3CA		0,35
chr1:115256536	C/C	C	NRAS		0,35
chr4:1807889	A/A	A	FGFR3		0,34
chr10:123279675	G/G	G	FGFR2		0,32
chr7:55221821	G/G	G	EGFR		0,32
chr7:55249070	A/A	A	EGFR ...(2)		0,3
chr4:55599339	A/A	A	KIT		0,3
chr3:178938935	A/A	A	PIK3CA		0,3
chr3:178916936	G/G	G	PIK3CA		0,3
chr2:29445213	A/A	A	ALK		0,3
chr15:90631839	T/T	T	IDH2		0,28

chr11:534285	C/C	C	HRAS		0,26
chr6:152419926	A/A	A	ESR1		0,25
chr1:115256529	T/T	T	NRAS		0,25
chr4:55599260	A/A	A	KIT		0,25
chr7:116423473	A/A	A	MET		0,25
chr3:178952019	C/C	C	PIK3CA		0,25
chr3:41266100	T/T	T	CTNNB1		0,25
chr17:37880220	T/T	T	ERBB2		0,25
chr17:37879903	C/C	C	ERBB2		0,25
chr10:123274794	T/T	T	FGFR2		0,25
chr7:140481403	C/C	C	BRAF		0,25
chr7:116423413	T/T	T	MET		0,25
chr7:55259502	A/A	A	EGFR		0,25
chr7:55211080	G/G	G	EGFR		0,25
chr4:55599261	G/G	G	KIT		0,25
chr3:178952081	G/G	G	PIK3CA		0,25
chr3:178952079	A/A	A	PIK3CA		0,25
chr3:178938860	A/A	A	PIK3CA		0,25
chr3:178922325	A/A	A	PIK3CA		0,25
chr3:178916890	C/C	C	PIK3CA		0,25
chr3:41266125	C/C	C	CTNNB1		0,25
chr3:41266104	G/G	G	CTNNB1		0,25
chr3:41266097	G/G	G	CTNNB1		0,25
chr2:29445270	A/A	A	ALK		0,25
chr1:11184573	G/G	G	MTOR		0,25
chr15:66727441	T/T	T	MAP2K1		0,24
chr10:43609949	G/G	G	RET		0,24
chr4:55593603	T/T	T	KIT		0,2
chr1:65310517	C/C	C	JAK1		0,2
chr11:533874	T/T	T	HRAS		0,2
chr11:533875	G/G	G	HRAS		0,2
chr3:41266136	T/T	T	CTNNB1		0,2
chr1:115258747	C/C	C	CSDE1 ...(	p.?	0,2
chr3:178952075	A/A	A	PIK3CA		0,2
chr3:178952065	G/G	G	PIK3CA		0,2
chr12:25398281	C/C	C	KRAS		0,2
chr10:123274774	A/A	A	FGFR2		0,2
chr10:123274773	C/C	C	FGFR2		0,2
chr7:140481411	C/C	C	BRAF		0,2
chr7:116412044	G/G	G	MET	p.?	0,2
chr4:55594221	A/A	A	KIT		0,2
chr3:178952090	G/G	G	PIK3CA		0,2

NFPE Prostate

chr5:176523562	A/A	C	FGFR4	p.?	100
chr2:29416572	C/C	T	ALK	p.Ile1461Va	99,95
chr5:176517326	C/C	T	FGFR4	p.?	99,85
chr4:55141055	G/G	A	PDGFRA	p.Pro567=	99,85
chr5:176523597	G/G	A	FGFR4	p.?	99,71
chr4:1807894	A/A	G	FGFR3	p.Thr651=	99,67
chr4:55566266	A/A	G	KIT	p.?	99,64
chr12:25386063	A/A	C	KRAS	p.?	99,55
chr5:176517797	T/T	C	FGFR4	p.Pro136Le	99,4
chr10:43613843	G/T	G	RET	p.Leu769=	51,6
chr7:55249063	G/A	G	EGFR ...(2	p.Gln787=	51,55
chr12:25389182	A/G	A	KRAS	p.?	51,13
chr5:112043384	T/G	T	APC		50,13
chr12:25400206	G/T	G	KRAS	p.?	50,09
chr3:178922274	C/A	C	PIK3CA	p.?	50,05
chr12:25386940	C/T	C	KRAS	p.?	49,27
chr4:55529199	T/TA	T	KIT	p.?	47,89
chr5:176517985	A/G	A	FGFR4	p.Ala161=	34,09
chr3:178952075	A/A	A	PIK3CA		0,45
chr4:1806099	A/A	A	FGFR3		0,44
chr3:12645699	G/G	G	RAF1		0,4
chr7:116411990	C/C	C	MET		0,4
chr3:41266104	G/G	G	CTNNB1		0,38
chr19:17945969	C/C	C	JAK3		0,35
chr6:152419926	A/A	A	ESR1		0,35
chr3:178952091	G/G	G	PIK3CA		0,35
chr4:55593661	T/T	T	KIT		0,31
chr12:56482341	G/G	G	ERBB3		0,3
chr7:55221821	G/G	G	EGFR		0,3
chr3:178952079	A/A	A	PIK3CA		0,3
chr3:178952018	A/A	A	PIK3CA		0,3
chr3:178916945	A/A	A	PIK3CA		0,3
chr1:115256536	C/C	C	NRAS		0,3
chr3:178936082	G/G	G	PIK3CA		0,25
chr12:25398282	C/C	C	KRAS		0,25
chr7:55211079	A/A	A	EGFR		0,25
chr7:116423474	T/T	T	MET		0,25
chr3:178922325	A/A	A	PIK3CA		0,25
chr7:140453145	A/A	A	BRAF		0,25
chr7:116423475	G/G	G	MET		0,25
chr3:178938860	A/A	A	PIK3CA		0,25
chr3:178936083	A/A	A	PIK3CA		0,25
chr3:178916726	G/G	G	PIK3CA		0,25
chr4:1806153	C/C	C	FGFR3		0,22
chr3:41266136	T/T	T	CTNNB1		0,22
chr3:41266109	C/C	C	CTNNB1		0,22
chr9:80409488	T/T	T	GNAQ		0,2
chr7:140453136	A/A	A	BRAF		0,2
chr7:55249004	A/A	A	EGFR ...(2)		0,2
chr1:115256529	T/T	T	NRAS		0,2
chr7:140453134	T/T	T	BRAF		0,2
chr3:178952072	A/A	A	PIK3CA		0,2
chr3:178952019	C/C	C	PIK3CA		0,2
chr2:29445213	A/A	A	ALK		0,2
chrX:66943543	C/C	C	AR		0,2
chr17:37881617	G/G	G	ERBB2		0,2
chr17:37868207	T/T	T	ERBB2		0,2
chr15:90631934	C/C	C	IDH2		0,2

chr12:25378562	C/C	C	KRAS	0,2
chr10:123279675	G/G	G	FGFR2	0,2
chr10:123274774	A/A	A	FGFR2	0,2
chr10:123274773	C/C	C	FGFR2	0,2
chr10:43617397	C/C	C	RET	0,2
chr10:43609102	T/T	T	RET	0,2
chr7:140481403	C/C	C	BRAF	0,2
chr7:116423414	A/A	A	MET	0,2
chr7:55249070	A/A	A	EGFR ...(2)	0,2
chr4:55599347	G/G	G	KIT	0,2
chr4:55599332	G/G	G	KIT	0,2
chr3:178952085	A/A	A	PIK3CA	0,2
chr3:178952084	C/C	C	PIK3CA	0,2
chr3:178921548	G/G	G	PIK3CA	0,2
chr3:178916725	C/C	C	PIK3CA	0,2
chr2:29432664	C/C	C	ALK	0,2
chr1:11188078	C/C	C	MTOR	0,2
chr15:66727441	T/T	T	MAP2K1	0,19
chr3:41266103	G/G	G	CTNNB1	0,16
chr3:41266113	C/C	C	CTNNB1	0,16
chr3:41266100	T/T	T	CTNNB1	0,16
chr3:41266097	G/G	G	CTNNB1	0,16
chr1:65310517	C/C	C	JAK1	0,15
chr4:55593609	G/G	G	KIT	0,15
chr4:55593603	T/T	T	KIT	0,15
chr4:55599260	A/A	A	KIT	0,15
chr19:17949164	T/T	T	JAK3	0,15
chr12:56481660	C/C	C	ERBB3	0,15
chrX:66943549	T/T	T	AR	0,15
chr15:90631935	G/G	G	IDH2	0,15
chr15:90631839	T/T	T	IDH2	0,15
chr10:123279674	G/G	G	FGFR2	0,15
chr7:128845572	G/G	G	SMO	0,15
chr4:1803565	G/G	G	FGFR3	0,15
chr3:178952088	A/A	A	PIK3CA	0,15
chr3:178952049	C/C	C	PIK3CA	0,15
chr3:178947826	T/T	T	PIK3CA	0,15
chr1:115252204	C/C	C	NRAS	0,15
chr1:11189845	G/G	G	MTOR	0,15
chr17:37880220	T/T	T	ERBB2	0,15
chr17:37879658	G/G	G	ERBB2	0,15
chr15:66729163	C/C	C	MAP2K1	0,15

FFPE Prostate

chr5:176523597	G/G	A	FGFR4	p.?	100
chr5:176523562	A/A	C	FGFR4	p.?	100
chr4:55141055	G/G	A	PDGFRA	p.Pro567=	99,95
chr4:1807894	A/A	G	FGFR3	p.Thr651=	99,92
chr2:29416572	C/C	T	ALK	p.Ile1461Val	99,9
chr4:55566266	A/A	G	KIT	p.?	99,88
chr12:25386063	A/A	C	KRAS	p.?	99,6
chr5:176517797	T/T	C	FGFR4	p.Pro136Leu	99,56
chr5:176517326	C/C	T	FGFR4	p.?	99,06
chr3:178922274	C/A	C	PIK3CA	p.?	53,62
chr12:25386940	C/T	C	KRAS	p.?	52,43
chr10:43613843	G/T	G	RET	p.Leu769=	51,68
chr12:25389182	A/G	A	KRAS	p.?	50,43
chr7:55249063	G/A	G	EGFR ...(2	p.Gln787=	48,6
chr5:112043384	T/G	T	APC		47,65
chr12:25400206	G/T	G	KRAS	p.?	47,09
chr4:55529199	T/TA	T	KIT	p.?	46,05
chr5:176517985	A/G	A	FGFR4	p.Ala161=	39,34
chr7:128845572	G/G	G	SMO		0,73
chr3:178952081	G/G	G	PIK3CA		0,65
chr1:115258745	C/C	C	CSDE1 ...(	p.?	0,65
chr7:55241677	G/G	G	EGFR		0,6
chr1:11188078	C/C	C	MTOR		0,57
chr3:178952091	G/G	G	PIK3CA		0,55
chr3:178947827	G/G	G	PIK3CA		0,55
chr3:178952084	C/C	C	PIK3CA		0,5
chr12:58145431	G/G	G	CDK4		0,47
chr4:55141036	T/T	T	PDGFRA		0,46
chrX:66943552	A/A	A	AR		0,45
chr7:116417463	C/C	C	MET		0,45
chr17:37881332	G/G	G	ERBB2		0,43
chr15:90631935	G/G	G	IDH2		0,42
chr3:178936082	G/G	G	PIK3CA		0,4
chr7:140481402	C/C	C	BRAF		0,4
chr2:209113112	C/C	C	IDH1		0,4
chr17:37881617	G/G	G	ERBB2		0,4
chr6:117638347	C/C	C	ROS1		0,4
chr3:178947826	T/T	T	PIK3CA		0,4
chr3:178922324	G/G	G	PIK3CA		0,4
chr3:12645699	G/G	G	RAF1		0,4
chr1:11184574	A/A	A	MTOR		0,4
chr7:55221821	G/G	G	EGFR		0,36
chr4:55593609	G/G	G	KIT		0,36
chr3:41266109	C/C	C	CTNNB1		0,36
chr3:41266098	A/A	A	CTNNB1		0,35
chr4:55599260	A/A	A	KIT		0,35
chr3:178952048	G/G	G	PIK3CA		0,35
chr3:178916944	A/A	A	PIK3CA		0,35
chr1:115252204	C/C	C	NRAS		0,35
chr9:5073770	G/G	G	JAK2		0,35
chr7:140453155	C/C	C	BRAF		0,3
chr17:37879903	C/C	C	ERBB2		0,3
chr10:43617397	C/C	C	RET		0,3
chr3:178952088	A/A	A	PIK3CA		0,3
chr3:178952079	A/A	A	PIK3CA		0,3
chr3:178928080	A/A	A	PIK3CA		0,3
chr3:178916890	C/C	C	PIK3CA		0,3
chr1:11184573	G/G	G	MTOR		0,3

chr3:41266112	T/T	T	CTNNB1	0,28
chr15:90631839	T/T	T	IDH2	0,28
chr12:58145430	C/C	C	CDK4	0,27
chr10:123274774	A/A	A	FGFR2	0,27
chr10:43609949	G/G	G	RET	0,25
chr17:37880997	G/G	G	ERBB2	0,25
chr7:116423473	A/A	A	MET	0,25
chr7:116423475	G/G	G	MET	0,25
chr7:140453134	T/T	T	BRAF	0,25
chr4:55599332	G/G	G	KIT	0,25
chr3:178952087	C/C	C	PIK3CA	0,25
chr3:178952082	C/C	C	PIK3CA	0,25
chr3:178952076	A/A	A	PIK3CA	0,25
chr3:178952072	A/A	A	PIK3CA	0,25
chr2:29445258	C/C	C	ALK	0,25
chr2:29432664	C/C	C	ALK	0,25
chr1:115256536	C/C	C	NRAS	0,25
chr7:128845571	C/C	C	SMO	0,24
chr11:533874	T/T	T	HRAS	0,23
chr11:534289	C/C	C	HRAS	0,22
chr4:55593603	T/T	T	KIT	0,2
chr7:140453133	T/T	T	BRAF	0,2
chr3:178952078	G/G	G	PIK3CA	0,2
chrX:66943543	C/C	C	AR	0,2
chr17:37880261	G/G	G	ERBB2	0,2
chr15:66729163	C/C	C	MAP2K1	0,2
chr12:56482341	G/G	G	ERBB3	0,2
chr12:56481660	C/C	C	ERBB3	0,2
chr12:56478817	G/G	G	ERBB3	0,2
chr12:25398285	C/C	C	KRAS	0,2
chr12:25378562	C/C	C	KRAS	0,2
chr10:123247516	T/T	T	FGFR2	0,2
chr7:140481412	C/C	C	BRAF	0,2
chr7:116423474	T/T	T	MET	0,2
chr7:116423413	T/T	T	MET	0,2
chr4:55599333	A/A	A	KIT	0,2
chr3:178952074	G/G	G	PIK3CA	0,2
chr3:178936070	G/G	G	PIK3CA	0,2
chr3:178922325	A/A	A	PIK3CA	0,2
chr3:178916945	A/A	A	PIK3CA	0,2
chr3:178916936	G/G	G	PIK3CA	0,2
chr2:29445270	A/A	A	ALK	0,2

NFPE Spleen

chr5:176523562	A/A	C	FGFR4	p.?	100
chr4:1807894	A/A	G	FGFR3	p.Thr651=	99,95
chr5:176517326	C/C	T	FGFR4	p.?	99,9
chr4:55141055	G/G	A	PDGFRA	p.Pro567=	99,9
chr2:29416572	C/C	T	ALK	p.Ile1461Val	99,9
chr4:55566266	A/A	G	KIT	p.?	99,89
chr5:176523597	G/G	A	FGFR4	p.?	99,85
chr5:176517797	T/T	C	FGFR4	p.Pro136Leu	99,5
chr12:25386063	A/A	C	KRAS	p.?	99,5
chr12:25389182	A/G	A	KRAS	p.?	53,1
chr12:25386940	C/T	C	KRAS	p.?	52,28
chr4:55529199	T/TA	T	KIT	p.?	51,94
chr7:55249063	G/A	G	EGFR ...(2	p.Gln787=	51,4
chr3:178922274	C/A	C	PIK3CA	p.?	50,98
chr12:25400206	G/T	G	KRAS	p.?	50,45
chr10:43613843	G/T	G	RET	p.Leu769=	48,12
chr5:112043384	T/G	T	APC		47,67
chr5:176517985	A/G	A	FGFR4	p.Ala161=	25,16
chr3:178952065	G/G	G	PIK3CA		0,55
chr10:123274773	C/C	C	FGFR2		0,5
chr3:178952074	G/G	G	PIK3CA		0,45
chr19:3118942	A/A	A	GNA11		0,4
chr3:178952048	G/G	G	PIK3CA		0,4
chr7:116423475	G/G	G	MET		0,4
chr4:55594258	T/T	T	KIT		0,4
chr1:11188078	C/C	C	MTOR		0,4
chr17:37880997	G/G	G	ERBB2		0,35
chr7:55249004	A/A	A	EGFR ...(2)		0,35
chr4:1803564	C/C	C	FGFR3		0,35
chr3:178916890	C/C	C	PIK3CA		0,35
chrX:66943543	C/C	C	AR		0,35
chr4:55141036	T/T	T	PDGFRA		0,35
chr12:25398284	C/C	C	KRAS		0,3
chr17:37880261	G/G	G	ERBB2		0,3
chr12:56481660	C/C	C	ERBB3		0,3
chr10:43617397	C/C	C	RET		0,3
chr3:178952082	C/C	C	PIK3CA		0,3
chr3:178916924	C/C	C	PIK3CA		0,3
chr19:3115013	G/G	G	GNA11		0,3
chr17:37880220	T/T	T	ERBB2		0,3
chr15:66727441	T/T	T	MAP2K1		0,3
chr12:25398285	C/C	C	KRAS		0,3
chr10:123274794	T/T	T	FGFR2		0,3
chr3:178916936	G/G	G	PIK3CA		0,3
chr3:178938935	A/A	A	PIK3CA		0,25
chr1:115258747	C/C	C	CSDE1 ...(	p.?	0,25
chr1:65310518	G/G	G	JAK1		0,25
chr17:37879658	G/G	G	ERBB2		0,25
chr12:58145430	C/C	C	CDK4		0,25
chr7:116423473	A/A	A	MET		0,25
chr4:55594221	A/A	A	KIT		0,25
chr1:115252203	G/G	G	NRAS		0,25
chr15:66729163	C/C	C	MAP2K1		0,25
chr12:56482341	G/G	G	ERBB3		0,25
chr12:25378561	G/G	G	KRAS		0,25
chr7:55241678	A/A	A	EGFR		0,25
chr3:178952084	C/C	C	PIK3CA		0,25
chr3:178952081	G/G	G	PIK3CA		0,25

chr3:178952018	A/A	A	PIK3CA		0,25
chr2:212488718	G/G	G	ERBB4		0,25
chr2:209113113	G/G	G	IDH1		0,25
chr2:29445270	A/A	A	ALK		0,25
chr1:11217230	C/C	C	MTOR		0,25
chr7:140453133	T/T	T	BRAF		0,2
chr7:55249005	G/G	G	EGFR ...(2)		0,2
chr19:17949164	T/T	T	JAK3		0,2
chr19:17945969	C/C	C	JAK3		0,2
chr12:58145431	G/G	G	CDK4		0,2
chr4:55599321	A/A	A	KIT		0,2
chr1:65310517	C/C	C	JAK1		0,2
chr15:90631934	C/C	C	IDH2		0,2
chr12:58145436	T/T	T	CDK4		0,2
chr12:56478854	G/G	G	ERBB3		0,2
chr10:123279674	G/G	G	FGFR2		0,2
chr10:43617416	T/T	T	RET		0,2
chr10:43617398	G/G	G	RET		0,2
chr10:43613839	A/A	A	RET		0,2
chr7:140481412	C/C	C	BRAF		0,2
chr7:140481411	C/C	C	BRAF		0,2
chr7:140481403	C/C	C	BRAF		0,2
chr7:140453154	T/T	T	BRAF		0,2
chr7:116412044	G/G	G	MET	p.?	0,2
chr7:55221822	C/C	C	EGFR		0,2
chr6:152419926	A/A	A	ESR1		0,2
chr3:178952090	G/G	G	PIK3CA		0,2
chr3:178952078	G/G	G	PIK3CA		0,2
chr3:178947826	T/T	T	PIK3CA		0,2
chr3:178936097	G/G	G	PIK3CA		0,2
chr3:178916891	G/G	G	PIK3CA		0,2
chr3:41266097	G/G	G	CTNNB1		0,2
chr1:11217231	A/A	A	MTOR		0,2
chr1:11189847	A/A	A	MTOR		0,2
chr1:11184573	G/G	G	MTOR		0,2
chr4:55593661	T/T	T	KIT		0,15
chr4:55593609	G/G	G	KIT		0,15
chr7:140481402	C/C	C	BRAF		0,15
chr1:115258744	C/C	C	CSDE1 ...(	p.?	0,15
chr3:178921548	G/G	G	PIK3CA		0,15
chr3:41266100	T/T	T	CTNNB1		0,15
chr1:11190804	C/C	C	MTOR		0,15

FFPE Spleen

chr5:176523597	G/G	A	FGFR4	p.?	100
chr5:176517326	C/C	T	FGFR4	p.?	99,85
chr2:29416572	C/C	T	ALK	p.Ile1461Val	99,85
chr12:25386063	A/A	C	KRAS	p.?	99,85
chr5:176523562	A/A	C	FGFR4	p.?	99,8
chr4:55141055	G/G	A	PDGFRA	p.Pro567=	99,77
chr4:1807894	A/A	G	FGFR3	p.Thr651=	99,7
chr4:55566266	A/A	G	KIT	p.?	99,68
chr5:176517797	T/T	C	FGFR4	p.Pro136Leu	99,55
chr12:25389182	A/G	A	KRAS	p.?	56,15
chr4:55529199	T/TA	T	KIT	p.?	51,14
chr12:25386940	C/T	C	KRAS	p.?	51,14
chr12:25400206	G/T	G	KRAS	p.?	50,72
chr5:112043384	T/G	T	APC		49,75
chr10:43613843	G/T	G	RET	p.Leu769=	47,8
chr3:178922274	C/A	C	PIK3CA	p.?	47,61
chr7:55249063	G/A	G	EGFR ...(2	p.Gln787=	44,74
chr5:176517985	A/G	A	FGFR4	p.Ala161=	32,86
chr17:37881332	G/G	G	ERBB2		0,95
chr3:41266109	C/C	C	CTNNB1		0,89
chr3:178952019	C/C	C	PIK3CA		0,6
chr3:178952087	C/C	C	PIK3CA		0,6
chr3:178916876	G/G	G	PIK3CA		0,55
chr7:140453137	C/C	C	BRAF		0,54
chr12:25378561	G/G	G	KRAS		0,5
chr17:37868208	C/C	C	ERBB2		0,47
chr1:162724598	C/C	C	DDR2		0,47
chr1:11190804	C/C	C	MTOR		0,47
chr10:43617398	G/G	G	RET		0,45
chr1:115258747	C/C	C	CSDE1 ...(	p.?	0,45
chr15:90631935	G/G	G	IDH2		0,42
chr15:90631934	C/C	C	IDH2		0,42
chr3:41266097	G/G	G	CTNNB1		0,41
chr3:41266104	G/G	G	CTNNB1		0,41
chr1:11188078	C/C	C	MTOR		0,41
chr3:178936082	G/G	G	PIK3CA		0,4
chr11:534289	C/C	C	HRAS		0,4
chr12:25398284	C/C	C	KRAS		0,4
chr3:178952065	G/G	G	PIK3CA		0,4
chr12:56481659	G/G	G	ERBB3		0,4
chr12:56478817	G/G	G	ERBB3		0,4
chr7:140481411	C/C	C	BRAF		0,4
chr4:55593660	C/C	C	KIT		0,4
chr3:178936098	A/A	A	PIK3CA		0,35
chr7:55221821	G/G	G	EGFR		0,32
chr10:123274773	C/C	C	FGFR2		0,31
chr6:152419926	A/A	A	ESR1		0,31
chr17:37879903	C/C	C	ERBB2		0,3
chr12:56482341	G/G	G	ERBB3		0,3
chr7:116423475	G/G	G	MET		0,3
chr3:178952081	G/G	G	PIK3CA		0,3
chr3:178952048	G/G	G	PIK3CA		0,3
chr3:178938935	A/A	A	PIK3CA		0,3
chr3:178938934	G/G	G	PIK3CA		0,3
chr3:178921549	T/T	T	PIK3CA		0,3
chr3:178921548	G/G	G	PIK3CA		0,3
chr3:178916854	G/G	G	PIK3CA		0,3
chr2:29443613	C/C	C	ALK		0,3

chr1:11184574	A/A	A	MTOR		0,3
chr7:116411990	C/C	C	MET		0,3
chr3:178916725	C/C	C	PIK3CA		0,28
chr14:105246551	C/C	C	AKT1		0,28
chrX:66943549	T/T	T	AR		0,25
chr9:80412493	C/C	C	GNAQ		0,25
chr2:212488718	G/G	G	ERBB4		0,25
chr17:37881616	C/C	C	ERBB2		0,25
chr4:55599261	G/G	G	KIT		0,25
chr3:178936083	A/A	A	PIK3CA		0,25
chr7:116412044	G/G	G	MET	p.?	0,24
chr19:3115012	C/C	C	GNA11		0,23
chr3:178922324	G/G	G	PIK3CA		0,23
chr12:58145431	G/G	G	CDK4		0,23
chr3:41266136	T/T	T	CTNNB1		0,21
chr10:123274774	A/A	A	FGFR2		0,21
chr3:41266106	A/A	A	CTNNB1		0,21
chr3:41266098	A/A	A	CTNNB1		0,21
chr7:55241677	G/G	G	EGFR		0,2
chr1:115256529	T/T	T	NRAS		0,2
chr12:25398282	C/C	C	KRAS		0,2
chrX:66943543	C/C	C	AR		0,2
chr11:534286	C/C	C	HRAS		0,2
chr10:43617416	T/T	T	RET		0,2
chr4:1803564	C/C	C	FGFR3		0,2
chr3:178936071	A/A	A	PIK3CA		0,2
chr19:3118942	A/A	A	GNA11		0,2
chr15:66729163	C/C	C	MAP2K1		0,2
chr10:43617397	C/C	C	RET		0,2
chr7:140481403	C/C	C	BRAF		0,2
chr7:128846398	C/C	C	SMO		0,2
chr7:55211080	G/G	G	EGFR		0,2
chr3:178952091	G/G	G	PIK3CA		0,2
chr3:178952082	C/C	C	PIK3CA		0,2
chr3:178936073	C/C	C	PIK3CA		0,2
chr3:178916924	C/C	C	PIK3CA		0,2
chr2:29445258	C/C	C	ALK		0,2
chr12:58145430	C/C	C	CDK4		0,17
chr7:55221822	C/C	C	EGFR		0,16
chr7:116417463	C/C	C	MET		0,16
chr10:123274795	A/A	A	FGFR2		0,16
chr7:128845571	C/C	C	SMO		0,15

NFPE Kidney

chr10:43613843	T/T	G	RET	p.Leu769=	100
chr5:176523562	A/A	C	FGFR4	p.?	100
chr2:29416572	C/C	T	ALK	p.Ile1461V	100
chr5:176517326	C/C	T	FGFR4	p.?	99,9
chr12:56477694	T/T	A	ERBB3	p.?	99,9
chr10:43615633	G/G	C	RET	p.Ser904=	99,9
chr4:55141055	G/G	A	PDGFRA	p.Pro567=	99,9
chr4:55529199	TA/TA	T	KIT	p.?	99,9
chr5:176523597	G/G	A	FGFR4	p.?	99,87
chr4:1807894	A/A	G	FGFR3	p.Thr651=	99,83
chr12:25386063	A/A	C	KRAS	p.?	99,75
chr5:176517797	T/T	C	FGFR4	p.Pro136Le	99,65
chr7:55249063	A/A	G	EGFR ...(2	p.Gln787=	99,6
chr4:55529160	GA/G	GA	KIT	p.?	84,93
chr12:25389182	A/G	A	KRAS	p.?	55,2
chr7:55259485	C/T	C	EGFR	p.Pro848Le	51,58
chr2:29416615	G/A	G	ALK	p.Thr1446=	51,55
chr8:128750322	G/A	G	MYC	p.?	51,13
chr2:29416366	G/C	G	ALK	p.Asp1529	50,1
chr3:178922274	C/A	C	PIK3CA	p.?	49,17
chr12:25386940	C/T	C	KRAS	p.?	49,07
chr12:25400206	G/T	G	KRAS	p.?	48,7
chr4:1809518	A/G	A	FGFR3	p.?	47,95
chr12:25364863	C/T	C	KRAS	p.?	33,6
chr5:176517985	A/G	A	FGFR4	p.Ala161=	26,39
chr4:55529169	AAACT/	AA/	KIT	p.?	10,36
chr12:25378561	G/G	G	KRAS		0,45
chr4:55593660	C/C	C	KIT		0,45
chr3:178916945	A/A	A	PIK3CA		0,4
chrX:66943549	T/T	T	AR		0,35
chr4:55599348	T/T	T	KIT		0,35
chr3:178936091	G/G	G	PIK3CA		0,35
chr4:1806119	G/G	G	FGFR3		0,3
chr4:55599321	A/A	A	KIT		0,3
chr3:178936098	A/A	A	PIK3CA		0,3
chr12:56481659	G/G	G	ERBB3		0,3
chr7:55241708	G/G	G	EGFR		0,3
chr4:55593609	G/G	G	KIT		0,25
chr7:140453136	A/A	A	BRAF		0,25
chr14:105246551	C/C	C	AKT1		0,25
chr12:25398281	C/C	C	KRAS		0,25
chr12:58145436	T/T	T	CDK4		0,25
chr12:58145430	C/C	C	CDK4		0,25
chr12:56482537	G/G	G	ERBB3		0,25
chr10:43609948	T/T	T	RET		0,25
chr10:43609102	T/T	T	RET		0,25
chr7:140453134	T/T	T	BRAF		0,25
chr7:55211079	A/A	A	EGFR		0,25
chr3:178947826	T/T	T	PIK3CA		0,25
chr3:178938934	G/G	G	PIK3CA		0,25
chr3:178916891	G/G	G	PIK3CA		0,25
chr3:41266101	C/C	C	CTNNB1		0,25
chr1:65310518	G/G	G	JAK1		0,25
chr1:11189847	A/A	A	MTOR		0,25
chr4:1807889	A/A	A	FGFR3		0,22
chr19:3118942	A/A	A	GNA11		0,2
chr7:140453145	A/A	A	BRAF		0,2
chr1:115258747	C/C	C	CSDE1 ...(	p.?	0,2

chr3:178952072	A/A	A	PIK3CA	0,2
chrX:66943585	A/A	A	AR	0,2
chr4:55594258	T/T	T	KIT	0,2
chr3:178952073	T/T	T	PIK3CA	0,2
chrX:66943552	A/A	A	AR	0,2
chr12:56478854	G/G	G	ERBB3	0,2
chr12:25398285	C/C	C	KRAS	0,2
chr12:25380276	T/T	T	KRAS	0,2
chr11:534289	C/C	C	HRAS	0,2
chr10:123279675	G/G	G	FGFR2	0,2
chr10:123247516	T/T	T	FGFR2	0,2
chr7:55221822	C/C	C	EGFR	0,2
chr3:178952088	A/A	A	PIK3CA	0,2
chr3:178952085	A/A	A	PIK3CA	0,2
chr3:178936092	A/A	A	PIK3CA	0,2
chr3:178916876	G/G	G	PIK3CA	0,2
chr3:178916725	C/C	C	PIK3CA	0,2
chr2:29445270	A/A	A	ALK	0,2
chr2:29445213	A/A	A	ALK	0,2
chr1:11184573	G/G	G	MTOR	0,2
chr15:90631935	G/G	G	IDH2	0,18
chr15:90631934	C/C	C	IDH2	0,18
chr4:55593603	T/T	T	KIT	0,15
chr4:55593661	T/T	T	KIT	0,15
chr3:41266137	C/C	C	CTNNB1	0,15
chr4:55593613	T/T	T	KIT	0,15
chr11:533874	T/T	T	HRAS	0,15
chr19:17949164	T/T	T	JAK3	0,15
chr1:115258744	C/C	C	CSDE1 ...(p.?	0,15
chr1:115256529	T/T	T	NRAS	0,15
chr10:43613839	A/A	A	RET	0,15
chr4:55599332	G/G	G	KIT	0,15
chr3:178936095	A/A	A	PIK3CA	0,15
chr19:4117551	A/A	A	MAP2K2	0,15
chr17:37880220	T/T	T	ERBB2	0,15
chr15:66729162	C/C	C	MAP2K1	0,15
chr12:56482341	G/G	G	ERBB3	0,15
chr12:56478817	G/G	G	ERBB3	0,15
chr12:25380283	C/C	C	KRAS	0,15
chr11:534286	C/C	C	HRAS	0,15
chr10:43617415	A/A	A	RET	0,15
chr10:43609949	G/G	G	RET	0,15

FFPE Kidney

chr5:176523597	G/G	A	FGFR4	p.?	100
chr5:176523562	A/A	C	FGFR4	p.?	100
chr4:55529199	TA/TA	T	KIT	p.?	100
chr10:43613843	T/T	G	RET	p.Leu769=	99,95
chr12:56477694	T/T	A	ERBB3	p.?	99,94
chr5:176517326	C/C	T	FGFR4	p.?	99,9
chr4:1807894	A/A	G	FGFR3	p.Thr651=	99,85
chr2:29416572	C/C	T	ALK	p.Ile1461V	99,8
chr7:55249063	A/A	G	EGFR ...(2	p.Gln787=	99,75
chr10:43615633	G/G	C	RET	p.Ser904=	99,72
chr5:176517797	T/T	C	FGFR4	p.Pro136Le	99,67
chr12:25386063	A/.	C	KRAS	p.?	93,53
chr4:55141055	G/.	A	PDGFRA	p.Pro567=	88,24
chr4:55529160	GA/G	GA	KIT	p.?	87,27
chr12:25386940	C/T	C	KRAS	p.?	62,59
chr12:25400206	G/T	G	KRAS	p.?	60,56
chr7:55259485	C/T	C	EGFR	p.Pro848Le	59,48
chr2:29416615	G/A	G	ALK	p.Thr1446=	48,05
chr12:25389182	A/G	A	KRAS	p.?	46,6
chr8:128750322	G/A	G	MYC	p.?	46,35
chr4:1809518	A/G	A	FGFR3	p.?	43,55
chr2:29416366	G/C	G	ALK	p.Asp1529	35,5
chr3:178922274	C/A	C	PIK3CA	p.?	33,86
chr12:25364863	C/T	C	KRAS	p.?	27,59
chr5:176517985	A/G	A	FGFR4	p.Ala161=	25,89
chrX:66941776	G/A	G	AR	p.Cys807T	23,13
chrX:66941817	G/A	G	AR	p.?	23,12
chrX:66941795	C/T	C	AR	p.Leu813=	14,63
chr12:56481574	C/T	C	ERBB3	p.?	14,41
chr12:25400273	G/A	G	KRAS	p.?	12,15
chr7:128845619	C/T	C	SMO	p.Pro306S	10,81
chr2:16084137	G/A	G	MYCN	p.?	10,58
chr12:56481631	C/T	C	ERBB3	p.Asn222=	10,54
chr10:43617331	C/T	C	RET	p.?	9,22
chr4:55529169	AAAC	AAA	KIT	p.?	9,2
chr17:37868180	G/A	G	ERBB2	p.?	9,11
chr6:117641070	G/A	G	ROS1	p.Ile1967=	8,46
chr3:178938956	C/T	C	PIK3CA	p.?	8,33
chr3:41266137	C/T	C	CTNNB1	p.Ser45Ph	8,3
chr7:140453155	C/T	C	BRAF	p.Asp594A	8,28
chr1:11184629	C/T	C	MTOR	p.Glu2196=	8,26
chrX:66915275	C/T	C	AR	p.?	8,23
chr17:41203062	G/A	G	BRCA1	p.?	8,12
chr4:55598854	G/A	G	KIT	p.?	7,93
chr7:116313571	C/T	C	MET	p.?	7,85
chrX:66917718	C/T	C	AR	p.?	7,77
chr3:178938859	C/T	C	PIK3CA	p.His701Ty	7,71
chr7:55221846	G/A	G	EGFR	p.?	7,46
chr12:25386080	C/T	C	KRAS	p.?	7,22
chr2:16084125	G/A	G	MYCN	p.?	7,2
chrX:70339217	C/T	C	MED12	p.?	7,15
chr12:58142078	G/A	G	CDK4 ...(3)	p.?, p.?	7,15
chr3:178916781	C/T	C	PIK3CA	p.Tyr56=	6,95
chr2:16084144	G/A	G	MYCN	p.?	6,92
chr9:80409495	C/T	C	GNAQ	p.Gly207Ar	6,86
chr15:66729173	G/A	G	MAP2K1	p.Val127=	6,8
chr12:56481611	G/A	G	ERBB3	p.Gly216Se	6,79
chr1:115252249	G/A	G	NRAS	p.His131Ty	6,77

chrX:66945191	C/T	C	AR	p.?	6,76
chr7:116412044	G/A	G	MET	p.?	6,69
chr2:29436955	G/A	G	ALK	p.?	6,65
chrX:66915203	C/T	C	AR	p.?	6,59
chr4:55141051	ACCC	GCC	PDGFRA	p.[Ser566A	6,58
chr1:65312399	G/A	G	JAK1	p.Ser640=	6,55
chr12:56482597	G/A	G	ERBB3	p.Val352M	6,54
chr4:55123765	C/T	C	PDGFRA	p.?	6,54
chr8:128752600	G/A	G	MYC	p.?	6,53
chr4:55573128	G/A	G	KIT	p.?	6,52
chrX:66866150	G/A	G	AR	p.?	6,51
chr12:25386060	TCAA	CCA	KRAS	p.?	6,47
chr17:41203132	G/A	G	BRCA1	p.Ile1760=	6,47
chr17:41203114	G/A	G	BRCA1	p.Ile1766=	6,47
chr17:41203137	G/A	G	BRCA1	p.?	6,46
chr7:116403218	C/T	C	MET	p.Leu845P	6,46
chr4:55140996	G/A	G	PDGFRA	p.?	6,36
chr2:29416160	G/A	G	ALK	p.Ala1598V	6,34
chr2:16084116	G/A	G	MYCN	p.?	6,28
chr12:58145361	C/T	C	CDK4	p.Gly47Glu	6,25
chr12:58145364	C/T	C	CDK4	p.Gly46Glu	6,24
chr7:116411969	G/A	G	MET	p.Ser1003A	6,23
chr4:55594190	G/A	G	KIT	p.Leu631=	6,2
chr2:29416430	C/T	C	ALK	p.Gly1508A	6,2
chrX:66866184	G/A	G	AR	p.?	6,15
chrX:66866214	G/A	G	AR	p.?	6,14
chr7:116412043	G/A	G	MET	p.Asp1028	6,13
chr12:25391244	C/T	C	KRAS	p.?	6,13
chr11:69466697	C/T	C	CCND1	p.?	6,12
chr4:55123795	G/A	G	PDGFRA	p.?	6,08
chr12:58144478	C/T	C	CDK4	p.Trp198Te	6,02
chrX:66945154	G/A	G	AR	p.?	6,02
chr7:140476699	G/A	G	BRAF	p.?	6,01
chr17:41203141	G/A	G	BRCA1	p.?	5,94
chr7:55221901	C/T	C	EGFR	p.?	5,93
chr17:41203052	G/A	G	BRCA1	p.?	5,88
chr5:176523333	C/T	C	FGFR4	p.Arg664Tr	5,85
chr8:38293046	C/T	C	FGFR1	p.?	5,85
chr11:69466664	C/T	C	CCND1	p.?	5,83
chr7:55242465	G/A	G	EGFR	p.Lys745=	5,83
chr1:162724599	G/A	G	DDR2	p.Arg124G	5,83
chr8:38293119	C/T	C	FGFR1	p.?	5,79
chrX:66866194	G/A	G	AR	p.?	5,77
chr17:37868223	G/A	G	ERBB2	p.Cys315T	5,74
chr7:55221838	G/A	G	EGFR	p.Lys294=	5,74
chr17:37868218	C/T	C	ERBB2	p.Leu313=	5,72
chr12:25391243	G/A	G	KRAS	p.?	5,72
chr12:58144647	C/T	C	CDK4	p.?	5,69
chr2:29436934	G/A	G	ALK	p.Ser1220I	5,69
chr7:140453104	C/T	C	BRAF	p.Glu611Ly	5,68
chr7:116417499	C/T	C	MET	p.His1124T	5,67
chr17:37872455	C/T	C	ERBB2	p.?	5,65
chr12:58144430	G/A	G	CDK4	p.?	5,64
chr12:58145365	C/T	C	CDK4	p.Gly46Arg	5,61
chr12:58144638	C/T	C	CDK4	p.?	5,56
chr2:29436939	G/A	G	ALK	p.Pro1218=	5,54
chr2:29497964	C/T	C	ALK	p.?	5,53
chr8:128753248	G/A	G	MYC	p.?	5,52
chr3:178922321	G/A	G	PIK3CA	p.Gly364Ar	5,41

chr4:55589789	G/A	G	KIT	p.Gly424As	5,35
chr1:162724511	C/T	C	DDR2	p.His95Tyr	5,31
chr12:25391170	G/A	G	KRAS	p.?	5,31
chr8:38287111	G/A	G	FGFR1	p.?	5,3
chr11:102248246	G/A	G	BIRC2	p.Arg413=	5,3
chr6:152419974	G/A	G	ESR1	p.Ser554As	5,3
chr6:117638380	G/A	G	ROS1	p.Pro2021S	5,25
chr4:1797235	G/A	G	FGFR3	p.?	5,24
chr1:11188171	G/A	G	MTOR	p.Pro1975S	5,2
chr4:55141054	CA/C/	CA	PDGFRA		5,18
chr1:11174439	G/A	G	MTOR	p.Asp2412	5,18
chr10:123312230	C/T	C	FGFR2	p.?	5,18
chr5:112175502	C/T	C	APC	p.Ser1404F	5,18
chr4:55594263	C/T	C	KIT	p.Leu656=	5,16
chr1:11174388	C/T	C	MTOR	p.Trp2429T	5,16
chr7:128849226	G/A	G	SMO	p.Arg485G	5,15
chr4:55529209	G/A	G	KIT	p.?	5,15
chr5:176518048	C/T	C	FGFR4	p.Ile182=	5,14
chr2:16084742	C/T	C	MYCN	p.?	5,11
chr12:25386948	G/A	G	KRAS	p.?	5,1
chr5:112175507	C/T	C	APC	p.Gln1406T	5,07
chr10:43617372	C/T	C	RET	p.?	5,02
chr7:116434529	G/A	G	MET	p.?	5,01
chr4:55529140	C/T	C	KIT	p.?	5,01
chr1:11188190	G/A	G	MTOR	p.?	5
chr1:11188273	G/A	G	MTOR	p.?	5
chr1:11188254	G/A	G	MTOR	p.?	5
chr7:116434510	C/T	C	MET	p.?	4,95
chr3:178936070	G/A	G	PIK3CA	p.Asp538A	4,92
chr10:123312256	C/T	C	FGFR2	p.?	4,9
chr1:11189880	G/A	G	MTOR	p.Leu1877I	4,9
chr12:56477681	C/T	C	ERBB3	p.Leu77=	4,88
chr5:176519569	G/A	G	FGFR4	p.?	4,88
chr10:123312221	C/T	C	FGFR2	p.?	4,88
chr19:17948786	C/T	C	JAK3	p.Glu552=	4,87
chrX:66866187	G/A	G	AR	p.?	4,82
chr12:56477655	C/T	C	ERBB3	p.Thr68Me	4,8
chr7:116415178	A/G	A	MET	p.?	4,8
chr7:55242458	C/T	C	EGFR	p.Ala743Va	4,8
chr11:69460795	G/A	G	CCND1	p.?	4,74
chr12:58145804	C/T	C	CDK4	p.?	4,74
chr12:58142058	G/A	G	CDK4 ...(3)	p.?, p.?	4,73
chr11:69458864	C/T	C	CCND1	p.?	4,7
chr8:128753197	G/A	G	MYC	p.Cys453T	4,69
chr11:69460799	C/T	C	CCND1	p.?	4,67
chr17:37881546	C/T	C	ERBB2	p.?	4,65
chr12:25364849	G/A	G	KRAS	p.?	4,64
chr4:55152088	C/T	C	PDGFRA	p.Ala840=	4,62
chrX:66915200	C/T	C	AR	p.?	4,6
chr17:37868215	C/T	C	ERBB2	p.Thr312=	4,6
chr2:29436957	G/A	G	ALK	p.?	4,59
chr8:38287110	G/A	G	FGFR1	p.?	4,55
chr1:11174378	C/T	C	MTOR	p.Asp2433	4,55
chr12:25386935	G/A	G	KRAS	p.?	4,55
chr8:38293017	C/T	C	FGFR1	p.?	4,54
chr9:80409480	C/T	C	GNAQ	p.Glu212Ly	4,54
chr7:128849155	C/T	C	SMO	p.Gly461=	4,51
chr4:1809471	G/A	G	FGFR3	p.?	4,51
chr1:115252213	C/T	C	NRAS	p.Glu143Ly	4,51

chr17:37880303	G/A	G	ERBB2	p.?	4,5
chr5:176523604	G/A	G	FGFR4	p.?	4,46
chr7:116417516	C/T	C	MET	p.Ser1129=	4,46
chr8:38287147	G/A	G	FGFR1	p.?	4,46
chr7:55249066	C/T	C	EGFR ...(2	p.Leu788=	4,45
chr7:128849169	C/T	C	SMO	p.Thr466Ile	4,43
chr12:25378571	C/T	C	KRAS	p.Glu143Ly	4,38
chr4:55097757	C/T	C	PDGFRA	p.?	4,33
chr17:37868158	C/T	C	ERBB2	p.?	4,33
chr10:43615679	C/T	C	RET	p.?	4,33
chr8:38271532	C/T	C	FGFR1	p.Met763Ile	4,3
chr4:55152140	C/T	C	PDGFRA	p.?	4,3
chr2:29443679	C/T	C	ALK	p.Val1180Il	3,5
chr3:178952090	G/A	G	PIK3CA	p.Gly1049S	3,3
chr2:29443631	G/A	G	ALK	p.Leu1196=	3
chr10:43609941	C/T	C	RET	p.Asp631=	2,38
chr7:55249071	C/T	C	EGFR ...(2	p.Thr790M	2,25
chr3:178916924	C/C	C	PIK3CA		1,85
chr7:140481402	C/C	C	BRAF		1,7
chrX:66943543	C/C	C	AR		1,4
chr1:115258747	C/C	C	CSDE1 ...(	p.?	1,36
chr19:4117551	A/A	A	MAP2K2		1,25
chr4:1806153	C/C	C	FGFR3		0,8
chr1:115258748	C/C	C	CSDE1 ...(	p.?	0,8

NFPE Liver

chr12:56477694	T/T	A	ERBB3	p.?	100
chr4:55529199	TA/TA	T	KIT	p.?	100
chr7:140476936	A/A	G	BRAF	p.?	99,95
chr7:55249063	A/A	G	EGFR ...(2	p.Gln787=	99,95
chr2:29416572	C/C	T	ALK	p.Ile1461Va	99,95
chr10:43613843	T/T	G	RET	p.Leu769=	99,95
chr5:176523562	A/A	C	FGFR4	p.?	99,95
chr5:176517326	C/C	T	FGFR4	p.?	99,8
chr4:55141055	G/G	A	PDGFRA	p.Pro567=	99,7
chr4:55097835	C/C	G	PDGFRA	p.?	99,7
chr4:1807894	A/A	G	FGFR3	p.Thr651=	99,65
chr12:25386063	A/A	C	KRAS	p.?	99,4
chr5:176517797	T/T	C	FGFR4	p.Pro136Le	99,3
chr3:182672831	CT/C	CT	DCUN1D1	p.?	77,67
chr4:55529160	G/.	GAA	KIT	p.?	70,04
chr3:178922274	C/A	C	PIK3CA	p.?	52,4
chr8:38271466	C/T	C	FGFR1	p.Leu785=	51,9
chr4:55133726	T/G	T	PDGFRA	p.Gly313=	50,85
chr4:55152040	C/T	C	PDGFRA	p.Val824=	50,55
chr12:58144665	C/T	C	CDK4	p.?	50,23
chr5:176523597	A/G	A	FGFR4	p.?	50,2
chr10:43615633	C/G	C	RET	p.Ser904=	48,52
chr4:55566266	G/A	G	KIT	p.?	48,42
chr11:69461995	T/C	T	CCND1	p.?	46,65
chr17:41216021	G/A	G	BRCA1	p.?	46,17
chr4:55598903	A/T	A	KIT	p.?	45,4
chr7:55199044	CT/C	CT	EGFR	p.?	37,56
chr12:25364863	C/T	C	KRAS	p.?	31,82
chr4:55529161	G/.	A	KIT	p.?	24,37
chr5:176517985	A/G	A	FGFR4	p.Ala161=	23,95
chr3:182672845	TGA/(	TGA	DCUN1D1	p.?	19,75
chr3:178916891	G/G	G	PIK3CA		0,7
chr3:178916854	G/G	G	PIK3CA		0,6
chr3:41266103	G/G	G	CTNNB1		0,55
chr1:65310518	G/G	G	JAK1		0,55
chr3:178916945	A/A	A	PIK3CA		0,45
chr7:55259502	A/A	A	EGFR		0,4
chr19:3118942	A/A	A	GNA11		0,4
chr17:37879903	C/C	C	ERBB2		0,4
chr12:56478817	G/G	G	ERBB3		0,4
chr11:534289	C/C	C	HRAS		0,4
chr7:116423475	G/G	G	MET		0,4
chr7:116417463	C/C	C	MET		0,4
chr3:178952090	G/G	G	PIK3CA		0,4
chr3:41266136	T/T	T	CTNNB1		0,35
chr1:65310517	C/C	C	JAK1		0,35
chr7:116417464	A/A	A	MET		0,35
chr4:55599348	T/T	T	KIT		0,35
chr4:55599321	A/A	A	KIT		0,35
chr2:29445258	C/C	C	ALK		0,35
chr3:41266137	C/C	C	CTNNB1		0,3
chr12:56481659	G/G	G	ERBB3		0,3
chr1:162724598	C/C	C	DDR2		0,3
chr7:140481411	C/C	C	BRAF		0,3
chr7:116423473	A/A	A	MET		0,3
chr7:116412044	G/G	G	MET	p.?	0,3
chr3:178952018	A/A	A	PIK3CA		0,3
chr3:41266097	G/G	G	CTNNB1		0,3

chr19:17949164	T/T	T	JAK3	0,25
chr12:25398284	C/C	C	KRAS	0,25
chr4:1808331	G/G	G	FGFR3	0,25
chr7:116423474	T/T	T	MET	0,25
chr3:178952079	A/A	A	PIK3CA	0,25
chr2:29445213	A/A	A	ALK	0,25
chr19:4117551	A/A	A	MAP2K2	0,25
chr12:25398285	C/C	C	KRAS	0,25
chr12:25380276	T/T	T	KRAS	0,25
chr12:25378562	C/C	C	KRAS	0,25
chr10:43617398	G/G	G	RET	0,25
chr4:1803564	C/C	C	FGFR3	0,25
chr3:178952088	A/A	A	PIK3CA	0,25
chr3:178928080	A/A	A	PIK3CA	0,25
chr3:178916924	C/C	C	PIK3CA	0,25
chr2:29445270	A/A	A	ALK	0,25
chr1:11189847	A/A	A	MTOR	0,25
chr17:37880997	G/G	G	ERBB2	0,2
chr1:115256529	T/T	T	NRAS	0,2
chr11:533874	T/T	T	HRAS	0,2
chr7:55211080	G/G	G	EGFR	0,2
chr4:1806120	G/G	G	FGFR3	0,2
chr1:11217231	A/A	A	MTOR	0,2
chrX:66943549	T/T	T	AR	0,2
chr15:66727441	T/T	T	MAP2K1	0,2
chr12:25380283	C/C	C	KRAS	0,2
chr12:25378561	G/G	G	KRAS	0,2
chr10:123279675	G/G	G	FGFR2	0,2
chr10:43609948	T/T	T	RET	0,2
chr10:43609097	G/G	G	RET	0,2
chr7:116411990	C/C	C	MET	0,2
chr4:55594258	T/T	T	KIT	0,2
chr4:1807889	A/A	A	FGFR3	0,2
chr3:178938935	A/A	A	PIK3CA	0,2
chr3:178936095	A/A	A	PIK3CA	0,2
chr3:12645699	G/G	G	RAF1	0,2
chr2:209113113	G/G	G	IDH1	0,2
chr4:55593603	T/T	T	KIT	0,15
chr7:55249070	A/A	A	EGFR ...(2)	0,15
chr3:178952073	T/T	T	PIK3CA	0,15
chr3:178916729	A/A	A	PIK3CA	0,15
chr2:29443696	A/A	A	ALK	

FFPE Liver

chr5:176523562	A/A	C	FGFR4	p.?	100
chr5:176517797	T/T	C	FGFR4	p.Pro136Leu	100
chr5:176517326	C/C	T	FGFR4	p.?	100
chr4:55529199	TA/TA	T	KIT	p.?	100
chr2:29416572	C/C	T	ALK	p.Ile1461Val	100
chr10:43613843	T/T	G	RET	p.Leu769=	99,93
chr4:55097835	C/C	G	PDGFRA	p.?	99,88
chr7:140476936	A/A	G	BRAF	p.?	99,8
chr4:1807894	A/A	G	FGFR3	p.Thr651=	99,75
chr7:55249063	A/A	G	EGFR ...(2	p.Gln787=	99,64
chr12:25386063	A/A	C	KRAS	p.?	99,6
chr4:55141055	G/G	A	PDGFRA	p.Pro567=	95,76
chr12:56477694	T/.	A	ERBB3	p.?	92,53
chr4:55529160	G/.	GAA	KIT	p.?	90,26
chr4:55598912	G/A	G	KIT	p.?	85,22
chr3:182672831	CT/C	CT	DCUN1D1	p.?	73,64
chr4:55566266	G/A	G	KIT	p.?	66,19
chr11:69461995	T/C	T	CCND1	p.?	60,88
chr12:58144665	C/T	C	CDK4	p.?	59,96
chr3:178922274	C/A	C	PIK3CA	p.?	58,97
chr4:55141035	G/A	G	PDGFRA	p.Val561Ile	53,57
chr4:55141065	G/A	G	PDGFRA	p.Glu571Lys	50,9
chr5:176523597	A/G	A	FGFR4	p.?	48,3
chr17:41216021	G/A	G	BRCA1	p.?	45,42
chr7:55199044	CT/C	CT	EGFR	p.?	44,44
chrX:66941756	C/T	C	AR	p.Ile800=	33,55
chr10:43615633	C/G	C	RET	p.Ser904=	33,2
chr4:1809251	C/T	C	FGFR3	p.?	31,91
chr2:29436861	G/A	G	ALK	p.His1244=	31,41
chr9:80409394	C/T	C	GNAQ	p.Val240=	31,33
chr8:38287093	G/A	G	FGFR1	p.?	30,36
chr7:140476734	G/A	G	BRAF	p.Arg558Ter	27,59
chr12:58145365	C/T	C	CDK4	p.Gly46Arg	27,22
chr5:176517985	A/G	A	FGFR4	p.Ala161=	26,05
chr2:16083924	G/A	G	MYCN	p.?	25,83
chr2:16083938	G/A	G	MYCN	p.?	25,76
chr12:25378630	C/T	C	KRAS	p.Arg123Lys	25,75
chr11:102238934	C/T	C	BIRC2	p.?	25,52
chr12:25364863	C/T	C	KRAS	p.?	25,07
chrX:66915214	G/A	G	AR	p.?	24,68
chr12:25386112	G/A	G	KRAS	p.?	24,1
chr3:178938864	G/A	G	PIK3CA	p.Leu702=	23,4
chr2:29416348	C/T	C	ALK	p.Leu1535=	23,35
chr7:55242438	G/A	G	EGFR	p.Glu736=	23,34
chr3:178938934	G/A	G	PIK3CA	p.Glu726Lys	23,07
chr7:55259475	G/A	G	EGFR	p.Val845Met	21,93
chr7:55221800	G/A	G	EGFR	p.Glu282Lys	21,65
chr8:38271541	C/T	C	FGFR1	p.Leu760=	21,17
chr6:117641099	C/T	C	ROS1	p.Glu1958Lys	20,86
chr1:65312308	C/T	C	JAK1	p.?	20,07
chr12:25391220	G/A	G	KRAS	p.?	19,61
chr12:25378605	C/T	C	KRAS	p.Gln131=	19,31
chr7:116415138	G/A	G	MET	p.Val1096Met	19,18
chr2:29416085	C/T	C	ALK	p.?	18,94
chr8:128752650	C/T	C	MYC	p.Gln271Ter	18,93
chr19:17945997	C/T	C	JAK3	p.Val648Ile	18,79
chr19:3115080	G/A	G	GNA11	p.?	18,71
chr4:1809239	G/A	G	FGFR3	p.?	18,41

chr2:16083966	C/T	C	MYCN	p.?	18,39
chrX:70339170	C/T	C	MED12	p.?	18,33
chr4:1797260	G/A	G	FGFR3	p.?	17,92
chr1:115252267	C/T	C	NRAS	p.Val125Ile	17,84
chr17:37872436	C/T	C	ERBB2	p.?	17,61
chr1:11188172	G/A	G	MTOR	p.Tyr1974=	17,51
chr1:11188244	G/A	G	MTOR	p.?	17,41
chr7:92424662	G/A	G	CDK6	p.?	17,15
chrX:70339174	CC/TT	CC	MED12	p.?	16,94
chr7:116434521	C/T	C	MET	p.?	16,79
chr19:17948801	C/T	C	JAK3	p.Glu547=	16,39
chr12:25364764	G/A	G	KRAS	p.?	16,35
chr4:1809528	C/T	C	FGFR3	p.?	16,34
chr3:182696949	C/T	C	DCUN1D1	p.?	16,21
chr4:1809259	C/T	C	FGFR3	p.?	16,19
chr19:17948762	G/A	G	JAK3	p.Ala560=	16,19
chr1:11188189	G/A	G	MTOR	p.?	16,13
chr4:1809277	C/T	C	FGFR3	p.?	16,06
chr6:152419928	C/T	C	ESR1	p.Leu539=	16,03
chr3:182672845	TGA/(	TGA	DCUN1D1	p.?	15,96
chr12:58144892	T/C	T	CDK4	p.?	15,96
chr7:116313560	G/A	G	MET	p.?	15,57
chr12:25391183	G/A	G	KRAS	p.?	15,42
chr12:25391242	G/A	G	KRAS	p.?	15,31
chr7:55242537	G/A	G	EGFR	p.?	15,29
chr10:43609883	C/T	C	RET	p.?	15,11
chr12:58144581	C/T	C	CDK4	p.?	15,09
chr7:116403189	C/T	C	MET	p.Thr835Ile	14,85
chr10:123284112	C/T	C	FGFR2	p.?	14,67
chr3:182693406	G/A	G	DCUN1D1	p.?	14,53
chr3:182693382	G/A	G	DCUN1D1	p.?	14,5
chr7:140482876	G/A	G	BRAF	p.Pro420Leu	14,43
chr7:140482843	G/A	G	BRAF	p.Ser431Phe	14,39
chr4:55598903	A/T	A	KIT	p.?	14,29
chr17:41203099	G/A	G	BRCA1	p.Pro1771=	14,07
chr1:11190876	C/T	C	MTOR	p.?	13,97
chr17:37872411	C/T	C	ERBB2	p.?	13,81
chr2:16084057	G/A	G	MYCN	p.?	13,75
chr17:37872455	C/T	C	ERBB2	p.?	13,74
chr3:178936082	G/A	G	PIK3CA	p.Glu542Lys	13,66
chr7:55221889	G/A	G	EGFR	p.?	13,61
chr8:38271758	G/A	G	FGFR1	p.Pro731Ser	13,53
chr3:178936148	G/A	G	PIK3CA	p.?	13,49
chr3:178936123	G/A	G	PIK3CA	p.?	13,48
chr1:162724523	C/T	C	DDR2	p.Leu99=	13,46
chr11:533838	C/T	C	HRAS	p.Arg73His	13,4
chr17:41203160	G/A	G	BRCA1	p.?	13,39
chrX:66866189	G/A	G	AR	p.?	13,28
chr17:29556993	G/A	G	NF1	p.?	13,26
chr7:55221906	G/A	G	EGFR	p.?	13,18
chr5:176524283	C/T	C	FGFR4	p.?	12,91
chr11:102237767	C/T	C	BIRC2	p.?	12,71
chr12:25364800	C/T	C	KRAS	p.?	12,65
chr1:11184640	G/A	G	MTOR	p.Arg2193Cys	12,49
chr4:1801128	G/A	G	FGFR3	p.Arg86His	12,46
chr17:29422265	C/T	C	MIR4733 ..	p.?	12,22
chr5:176523350	G/A	G	FGFR4	p.Gln669=	12,15
chr4:55593437	G/A	G	KIT	p.Val532Ile	11,97
chr1:11189850	C/T	C	MTOR	p.Gly1887Ser	11,91

chr7:116313572	C/T	C	MET	p.?	11,91
chrX:66874528	C/T	C	AR	p.?	11,82
chr1:11189890	C/T	C	MTOR	p.Leu1873=	11,78
chr8:38282216	C/A	C	FGFR1	p.Glu280Asp	11,72
chr17:37883142	G/A	G	ERBB2 ...(	p.Gly1015=	11,47
chr12:58145755	C/T	C	CDK4	p.?	11,32
chr19:17948843	G/A	G	JAK3	p.Thr533=	11,31
chr2:29443596	G/A	G	ALK	p.Phe1207=	11,15
chr17:41258587	A/G	A	BRCA1	p.?	11,08
chr3:178921476	G/A	G	PIK3CA	p.Gly320Arg	11,08
chr11:69461064	C/T	C	CCND1	p.?	11,02
chr10:43609966	G/A	G	RET	p.Ala640Thr	10,86
chrX:66938942	C/T	C	AR	p.?	10,82
chr1:11190808	G/A	G	MTOR	p.Asn1797=	10,82
chr12:25400173	A/G	A	KRAS	p.?	10,78
chr17:41258541	C/T	C	BRCA1	p.Met48Ile	10,64
chr12:58143250	C/T	C	CDK4 ...(2)	p.Gly224Ser	10,59
chr2:212488747	C/T	C	ERBB4	p.Ser701Asn	10,54
chr2:29445428	C/T	C	ALK	p.Val1135=	10,45
chr4:55161902	G/A	G	PDGFRA	p.?	10,41
chr2:16084746	T/C	T	MYCN	p.?	10,36
chr10:43609092	G/A	G	RET	p.Glu616=	10,13
chr17:29657280	G/A	G	NF1	p.?	10,1
chr3:178921520	C/T	C	PIK3CA	p.Leu334=	10,09
chr10:43609107	G/A	G	RET	p.Glu621=	10,02
chr12:56477614	G/A	G	ERBB3	p.Glu54=	9,98
chr12:56481592	C/T	C	ERBB3	p.Ile209=	9,95
chr8:38315071	C/T	C	FGFR1	p.?	9,89
chr12:58144586	G/A	G	CDK4	p.?	9,77
chr4:55529161	G/.	A	KIT	p.?	9,74
chr11:102238628	C/T	C	BIRC2	p.?	9,66
chr12:56482544	C/T	C	ERBB3	p.Thr334Ile	9,66
chr1:11217217	C/T	C	MTOR	p.Leu1487=	9,65
chr5:176518106	G/A	G	FGFR4	p.?	9,63
chr7:116434483	G/A	G	MET	p.?	9,59
chr8:38285455	C/T	C	FGFR1	p.Arg233Lys	9,15
chr15:66774097	C/T	C	MAP2K1	p.Val191=	9,07
chr2:29443686	C/T	C	ALK	p.Gln1177=	8,94
chr1:11184543	G/A	G	MTOR	p.?	8,88
chr7:55242428	C/T	C	EGFR	p.Pro733Leu	8,87
chr4:55593495	C/T	C	KIT	p.?	8,87
chr10:43609939	G/A	G	RET	p.Asp631Asn	8,82
chr3:178921497	C/T	C	PIK3CA	p.Leu327Phe	8,81
chr7:92286911	C/T	C	CDK6	p.?	8,79
chr5:176517409	G/A	G	FGFR4	p.Ser37Asn	8,62
chr1:11217253	C/T	C	MTOR	p.Glu1475=	8,55
chr5:176523653	C/T	C	FGFR4	p.Ser688=	8,53
chr12:56482310	C/T	C	ERBB3	p.?	8,51
chr10:123247563	G/A	G	FGFR2	p.Ala643Val	8,5
chrX:66874572	C/T	C	AR	p.?	8,38
chr5:176519535	G/A	G	FGFR4	p.?	8,37
chr5:176523322	C/T	C	FGFR4	p.Ala660Val	8,31
chr4:1805828	C/T	C	FGFR3	p.?	8,26
chr17:37881035	C/T	C	ERBB2	p.Ile788=	8,22
chr6:152419994	G/A	G	ESR1	p.Glu561Lys	8,21
chr17:37880959	C/T	C	ERBB2	p.?	8,18
chr11:102248234	G/A	G	BIRC2	p.Leu409=	8,13
chr11:102237836	C/T	C	BIRC2	p.?	8,08
chr11:69461983	G/A	G	CCND1	p.?	8,03

chr17:37881554	G/A	G	ERBB2	p.?	7,99
chr17:29422363	C/T	C	MIR4733 ..	p.Ala12=	7,78
chrX:66866163	G/A	G	AR	p.?	7,77
chrX:66799248	G/A	G	AR	p.?	7,75
chrX:66866175	G/A	G	AR	p.?	7,68
chr8:128752032	G/A	G	MYC	p.?	7,65
chr8:38285504	G/A	G	FGFR1	p.Pro217Ser	7,65
chr1:11188070	C/T	C	MTOR	p.Gln2008=	7,62
chr8:128748887	C/T	C	MYC	p.?	7,61
chr4:55097803	C/T	C	PDGFRA	p.?	7,61
chr3:12645716	G/A	G	RAF1	p.Leu251=	7,6
chr7:140481430	C/T	C	BRAF	p.Gly460Arg	7,54
chr12:56477694	TGT/.	AGC	ERBB3	p.?	7,47
chr4:55589729	G/A	G	KIT	p.?	7,45
chrX:66866207	G/A	G	AR	p.?	7,39
chr7:92286913	C/T	C	CDK6	p.?	7,39
chrX:70349200	C/T	C	MED12	p.Asp1204=	7,15
chr15:66729197	C/T	C	MAP2K1	p.Ser135=	7,15
chr2:29420429	G/A	G	ALK	p.Pro1351Leu	7,06
chrX:66866159	G/A	G	AR	p.?	7,01
chr2:16085485	G/A	G	MYCN	p.?	7
chr4:55104307	C/T	C	PDGFRA	p.?	6,97
chr4:55589778	G/A	G	KIT	p.Arg420=	6,86
chr4:55589768	C/T	C	KIT	p.Thr417Ile	6,85
chr5:176517366	G/A	G	FGFR4	p.?	6,8
chrX:70349217	C/T	C	MED12	p.Ala1210Val	6,7
chr12:56478865	G/A	G	ERBB3	p.Gly107=	6,67
chr12:56478892	C/T	C	ERBB3	p.Ala116=	6,64
chr5:176517348	G/A	G	FGFR4	p.?	6,62
chr15:66729137	G/A	G	MAP2K1	p.Leu115=	6,56
chr2:212488749	G/A	G	ERBB4	p.Pro700=	6,43
chr4:55104335	C/T	C	PDGFRA	p.?	6,43
chr5:176523282	C/T	C	FGFR4	p.?	6,42
chr11:102238599	C/A	C	BIRC2	p.?	6,4
chr2:29497952	G/A	G	ALK	p.?	6,4
chr11:69458961	C/T	C	CCND1	p.?	6,3
chr17:41203137	G/A	G	BRCA1	p.?	6,25
chr12:58144947	A/T	A	CDK4	p.?	6,25
chr11:69466973	C/T	C	CCND1	p.?	6,25
chr3:41266078	G/A	G	CTNNB1	p.Trp25Ter	6,19
chr17:41203144	G/A	G	BRCA1	p.?	6,12
chr7:92383788	G/A	G	CDK6	p.?	6,1
chr1:11188022	G/A	G	MTOR	p.?	6,01
chr10:43613796	C/T	C	RET	p.?	6,01
chr11:534286	C/T	C	HRAS	p.Gly13Ser	6
chr17:37882892	C/T	C	ERBB2 ...(	p.Gln984Ter	5,94
chr7:140507793	C/T	C	BRAF	p.Val226=	5,9
chr1:11188050	C/T	C	MTOR	p.?	5,88
chr7:140434453	C/T	C	BRAF	p.Ala749Thr	5,88
chr7:92383765	C/T	C	CDK6	p.?	5,85
chr10:43613854	T/C	T	RET	p.Leu773Pro	5,81
chr11:69458918	C/T	C	CCND1	p.?	5,8
chrX:66915234	T/C	T	AR	p.?	5,79
chr17:41215993	G/A	G	BRCA1	p.?	5,65
chr12:58142909	C/T	C	CDK4 ...(3)	p.?, p.?	5,65
chr4:55144139	C/T	C	PDGFRA	p.Asn656=	5,64
chr11:534263	G/A	G	HRAS	p.Thr20=	5,6
chr10:123257959	C/T	C	FGFR2	p.?	5,6
chr7:116415185	T/C	T	MET	p.?	5,58

chr11:69458921	G/A	G	CCND1	p.?	5,55
chr11:102234245	G/A	G	BIRC2	p.?	5,54
chr2:209113117	T/C	T	IDH1	p.Ile130Met	5,52
chr3:178928095	C/T	C	PIK3CA	p.Pro458Leu	5,46
chr4:55161927	G/A	G	PDGFRA	p.?	5,46
chr7:55233045	G/A	G	EGFR	p.Val599Ile	5,45
chr3:182693319	G/A	G	DCUN1D1	p.?	5,42
chr4:55589731	C/T	C	KIT	p.?	5,4
chr11:69457869	C/T	C	CCND1	p.Ser90Leu	5,4
chr10:123279670	G/A	G	FGFR2	p.His254=	5,4
chr7:92273269	A/G	A	CDK6	p.?	5,39
chr4:1809235	G/T	G	FGFR3	p.?	5,38
chr11:69462019	G/A	G	CCND1	p.?	5,38
chr3:178927928	G/A	G	PIK3CA	p.?	5,34
chr4:55593666	T/C	T	KIT	p.Tyr578His	5,24
chr2:29445319	C/T	C	ALK	p.?	5,23
chr12:58142938	C/T	C	CDK4 ...(3)	p.?, p.?	5,2
chr2:16080731	G/A	G	MYCN ...(2	p.?	5,19
chr17:41215998	G/A	G	BRCA1	p.?	5,15
chr12:56478814	C/T	C	ERBB3	p.Ala90=	5,08
chr7:92383756	G/A	G	CDK6	p.?	5,05
chr7:140507822	G/T	G	BRAF	p.Leu217Ile	5,01
chr17:41215992	G/A	G	BRCA1	p.?	5
chr19:4117591	C/T	C	MAP2K2	p.Glu43=	4,98
chr11:534302	C/T	C	HRAS	p.Val7=	4,95
chr10:123279660	G/A	G	FGFR2	p.Leu258Phe	4,9
chr1:115258761	C/T	C	CSDE1 ...(	p.Val7=, p.?	4,9
chr15:66729225	C/T	C	MAP2K1	p.His145Tyr	4,88
chr17:41215986	C/T	C	BRCA1	p.?	4,8
chr12:25378557	C/T	C	KRAS	p.Lys147=	4,78
chrX:66917726	G/A	G	AR	p.?	4,77
chr7:55219934	C/T	C	EGFR	p.?	4,77
chr19:3118885	C/T	C	GNA11	p.?	4,65
chr11:69466993	C/T	C	CCND1	p.?	4,65
chr4:1806158	G/A	G	FGFR3	p.Val393Met	4,65
chr6:152419983	G/A	G	ESR1	p.Gly557Glu	4,62
chr7:116423476	G/A	G	MET	p.Ala1269Thr	4,6
chr12:56478805	C/T	C	ERBB3	p.Val87=	4,6
chr7:92292516	G/A	G	CDK6	p.?	4,55
chr7:140507787	C/T	C	BRAF	p.Glu228=	4,55
chr7:55233026	C/T	C	EGFR	p.Val592=	4,55
chr7:92383833	C/T	C	CDK6	p.?	4,5
chr2:16083284	G/A	G	MYCN	p.?	4,49
chr11:69457932	C/T	C	CCND1	p.Ser111Phe	4,45
chr19:17949128	G/T	G	JAK3	p.Gln505Lys	4,4
chr2:29432724	C/T	C	ALK	p.Cys1255Tyr	4,4
chr8:128749169	T/C	T	MYC	p.?	4,32
chr7:55242499	A/G	A	EGFR	p.Lys757Glu	3,52
chrX:66943543	C/T	C	AR	p.His875Tyr	3,35
chr12:25380275	T/T	T	KRAS		1,07
chr12:56481659	G/G	G	ERBB3		0,84
chr19:4117551	A/A	A	MAP2K2		0,68
chr4:55593661	T/T	T	KIT		0,49
chr4:55593610	T/T	T	KIT		0,49
chr7:55249070	A/A	A	EGFR ...(2)		0,45
chr7:116423413	T/T	T	MET		0,4
chr4:1807889	A/A	A	FGFR3		0,3
chr3:12645699	G/G	G	RAF1		0,3
chr7:55249004	A/A	A	EGFR ...(2)		0,27

chr7:55249071	C/C	C	EGFR ...(2)	0,27
chr19:3118942	A/A	A	GNA11	0,25
chr7:55211080	G/G	G	EGFR	0,25
chr7:55211079	A/A	A	EGFR	0,25
chr3:178938860	A/A	A	PIK3CA	0,25
chr3:178922363	T/T	T	PIK3CA	0,22

NFPE Uterus

chr10:43613843	T/T	G	RET	p.Leu769=	100
chr5:176523562	A/A	C	FGFR4	p.?	100
chr2:29416572	C/C	T	ALK	p.Ile1461Val	100
chr5:176517326	C/C	T	FGFR4	p.?	99,9
chr12:56477694	T/T	A	ERBB3	p.?	99,9
chr10:43615633	G/G	C	RET	p.Ser904=	99,9
chr4:55141055	G/G	A	PDGFRA	p.Pro567=	99,9
chr4:55529199	TA/TA	T	KIT	p.?	99,9
chr5:176523597	G/G	A	FGFR4	p.?	99,87
chr4:1807894	A/A	G	FGFR3	p.Thr651=	99,83
chr12:25386063	A/A	C	KRAS	p.?	99,75
chr5:176517797	T/T	C	FGFR4	p.Pro136Leu	99,65
chr7:55249063	A/A	G	EGFR ...(2	p.Gln787=	99,6
chr4:55529160	GA/G	GA	KIT	p.?	84,93
chr12:25389182	A/G	A	KRAS	p.?	55,2
chr7:55259485	C/T	C	EGFR	p.Pro848Leu	51,58
chr2:29416615	G/A	G	ALK	p.Thr1446=	51,55
chr8:128750322	G/A	G	MYC	p.?	51,13
chr2:29416366	G/C	G	ALK	p.Asp1529Glu	50,1
chr3:178922274	C/A	C	PIK3CA	p.?	49,17
chr12:25386940	C/T	C	KRAS	p.?	49,07
chr12:25400206	G/T	G	KRAS	p.?	48,7
chr4:1809518	A/G	A	FGFR3	p.?	47,95
chr12:25364863	C/T	C	KRAS	p.?	33,6
chr5:176517985	A/G	A	FGFR4	p.Ala161=	26,39
chr4:55529169	AAACT/(	AAAC	KIT	p.?	10,36
chr12:25378561	G/G	G	KRAS		0,45
chr4:55593660	C/C	C	KIT		0,45
chr3:178916945	A/A	A	PIK3CA		0,4
chrX:66943549	T/T	T	AR		0,35
chr4:55599348	T/T	T	KIT		0,35
chr3:178936091	G/G	G	PIK3CA		0,35
chr4:1806119	G/G	G	FGFR3		0,3
chr4:55599321	A/A	A	KIT		0,3
chr3:178936098	A/A	A	PIK3CA		0,3
chr12:56481659	G/G	G	ERBB3		0,3
chr7:55241708	G/G	G	EGFR		0,3
chr4:55593609	G/G	G	KIT		0,25
chr7:140453136	A/A	A	BRAF		0,25
chr14:105246551	C/C	C	AKT1		0,25
chr12:25398281	C/C	C	KRAS		0,25
chr12:58145436	T/T	T	CDK4		0,25
chr12:58145430	C/C	C	CDK4		0,25
chr12:56482537	G/G	G	ERBB3		0,25
chr10:43609948	T/T	T	RET		0,25
chr10:43609102	T/T	T	RET		0,25
chr7:140453134	T/T	T	BRAF		0,25
chr7:55211079	A/A	A	EGFR		0,25
chr3:178947826	T/T	T	PIK3CA		0,25
chr3:178938934	G/G	G	PIK3CA		0,25
chr3:178916891	G/G	G	PIK3CA		0,25
chr3:41266101	C/C	C	CTNNB1		0,25
chr1:65310518	G/G	G	JAK1		0,25
chr1:11189847	A/A	A	MTOR		0,25
chr4:1807889	A/A	A	FGFR3		0,22
chr19:3118942	A/A	A	GNA11		0,2
chr7:140453145	A/A	A	BRAF		0,2
chr1:115258747	C/C	C	CSDE1 ...(	p.?	0,2

chr3:178952072	A/A	A	PIK3CA	0,2
chrX:66943585	A/A	A	AR	0,2
chr4:55594258	T/T	T	KIT	0,2
chr3:178952073	T/T	T	PIK3CA	0,2
chrX:66943552	A/A	A	AR	0,2
chr12:56478854	G/G	G	ERBB3	0,2
chr12:25398285	C/C	C	KRAS	0,2
chr12:25380276	T/T	T	KRAS	0,2
chr11:534289	C/C	C	HRAS	0,2
chr10:12327967[illegible]	G/G	G	FGFR2	0,2
chr10:12324751[illegible]	T/T	T	FGFR2	0,2
chr7:55221822	C/C	C	EGFR	0,2
chr3:178952088	A/A	A	PIK3CA	0,2
chr3:178952085	A/A	A	PIK3CA	0,2
chr3:178936092	A/A	A	PIK3CA	0,2
chr3:178916876	G/G	G	PIK3CA	0,2
chr3:178916725	C/C	C	PIK3CA	0,2
chr2:29445270	A/A	A	ALK	0,2
chr2:29445213	A/A	A	ALK	0,2
chr1:11184573	G/G	G	MTOR	0,2
chr15:90631935	G/G	G	IDH2	0,18
chr15:90631934	C/C	C	IDH2	0,18
chr4:55593603	T/T	T	KIT	0,15
chr4:55593661	T/T	T	KIT	0,15
chr3:41266137	C/C	C	CTNNB1	0,15
chr4:55593613	T/T	T	KIT	0,15
chr11:533874	T/T	T	HRAS	0,15
chr19:17949164	T/T	T	JAK3	0,15
chr1:115258744	C/C	C	CSDE1 ...(p.?	0,15
chr1:115256529	T/T	T	NRAS	0,15
chr10:43613839	A/A	A	RET	0,15
chr4:55599332	G/G	G	KIT	0,15
chr3:178936095	A/A	A	PIK3CA	0,15
chr19:4117551	A/A	A	MAP2K2	0,15
chr17:37880220	T/T	T	ERBB2	0,15
chr15:66729162	C/C	C	MAP2K1	0,15
chr12:56482341	G/G	G	ERBB3	0,15
chr12:56478817	G/G	G	ERBB3	0,15
chr12:25380283	C/C	C	KRAS	0,15
chr11:534286	C/C	C	HRAS	0,15
chr10:43617415	A/A	A	RET	0,15
chr10:43609949	G/G	G	RET	0,15

FFPE Uterus

chr5:176523562	A/A	C	FGFR4	p,?	100
chr5:176517326	C/C	T	FGFR4	p,?	100
chr4:55529199	TA/TA	T	KIT	p,?	100
chr4:55141055	G/G	A	PDGFRA	p,Pro567=	100
chr3:178922274	A/A	C	PIK3CA	p,?	100
chr4:1807894	A/A	G	FGFR3	p,Thr651=	99,95
chr2:29416572	C/C	T	ALK	p,Ile1461V	99,9
chr12:25386063	A/A	C	KRAS	p,?	99,65
chr5:176517797	T/T	C	FGFR4	p,Pro136L	99,54
chr12:58144665	C/T	C	CDK4	p,?	66,03
chr10:43613843	G/T	G	RET	p,Leu769=	59,95
chr12:56477694	A/T	A	ERBB3	p,?	58,59
chr7:55249063	G/A	G	EGFR ,,,(2	p,Gln787=	53,23
chr4:1797741	T/C	T	FGFR3	p,?	49,87
chr7:92286918	A/G	A	CDK6	p,?	45,22
chr5:176523597	A/G	A	FGFR4	p,?	44,76
chr3:182672831	CT/C	CT	DCUN1D1	p,?	44,24
chr4:55152120	C/T	C	PDGFRA	p,Ser851L	40,5
chr4:55097835	G/C	G	PDGFRA	p,?	36,09
chr3:182672845	TGA/G	TGA	DCUN1D1	p,?	24,82
chr3:182696920	G/A	G	DCUN1D1	p,?	23,68
chr3:178936093	G/A	G	PIK3CA	p,Glu545=	23,08
chr4:55123734	G/A	G	PDGFRA	p,?	22,39
chr4:55573041	G/A	G	KIT	p,?	20,95
chr4:55573053	G/A	G	KIT	p,?	20,71
chr17:37868205	G/A	G	ERBB2	p,Gly309G	17,56
chr7:116339338	C/T	C	MET	p,Thr67Ile	17,45
chr17:37868189	C/T	C	ERBB2	p,Leu304P	17,28
chr5:176517985	A/G	A	FGFR4	p,Ala161=	17,24
chr17:37868214	C/T	C	ERBB2	p,Thr312Il	17,23
chr1:11189851	C/T	C	MTOR	p,Gln1886	17,02
chr1:65310483	G/A	G	JAK1	p,Ile735=	16,93
chr5:112173273	G/A	G	APC	p,Cys661T	16,67
chr2:29416101	G/A	G	ALK	p,Pro1618	16,67
chr6:117638353	C/T	C	ROS1	p,Glu2030	16,58
chr12:56482584	C/T	C	ERBB3	p,Asn347=	16,27
chr17:29556958	G/A	G	NF1	p,Ala986T	16,09
chr3:182693383	G/A	G	DCUN1D1	p,?	16,02
chr3:182693365	G/A	G	DCUN1D1	p,?	14,67
chr10:123350122	C/T	C	FGFR2	p,?	14,67
chr3:182693314	C/T	C	DCUN1D1	p,?	14,2
chr12:58142313	C/T	C	CDK4 ,,,(3)	p,?, p,Glu3	14,1
chr17:41276051	G/A	G	BRCA1	p,Ile21=	13,99
chrX:66941743	G/A	G	AR	p,Gly796G	13,95
chr7:116339441	C/T	C	MET	p,Cys101=	13,51
chr15:66729163	C/T	C	MAP2K1	p,Pro124L	13,31
chr12:56482578	G/A	G	ERBB3	p,Ser345=	13,16
chr5:112151209	G/A	G	APC	p,Met284Il	13,12
chr2:16084051	C/T	C	MYCN	p,?	12,79
chr2:16083982	G/A	G	MYCN	p,?	12,54
chr8:38283570	C/T	C	FGFR1	p,?	12,31
chr1:11190778	C/T	C	MTOR	p,Gln1807	12,17
chr12:25391227	C/T	C	KRAS	p,?	11,97
chr4:55104306	C/T	C	PDGFRA	p,?	11,93
chr2:16084906	C/T	C	MYCN	p,?	11,83
chr8:128753155	G/A	G	MYC	p,Arg439G	11,59
chrX:66874527	G/A	G	AR	p,?	11,58
chrX:66874543	G/A	G	AR	p,?	11,52

chr8:128752605	C/T	C	MYC	p,?	11
chr7:116417532	C/T	C	MET	p,?	10,96
chr3:182691239	G/A	G	DCUN1D1	p,?	10,81
chr12:25389188	G/A	G	KRAS	p,?	10,64
chr9:80409396	C/T	C	GNAQ	p,Val240M	10,58
chr15:90631882	G/A	G	IDH2	p,Ile157=	10,42
chr7:116434505	C/T	C	MET	p,?	10,41
chr8:38283680	C/T	C	FGFR1	p,Glu266=	10,38
chr17:37883195	C/T	C	ERBB2 ,,,(:	p,Pro1033I	10,32
chr1:65312310	C/T	C	JAK1	p,?	10,12
chr1:11174479	G/A	G	MTOR	p,Thr2399I	10,08
chr7:140482825	C/T	C	BRAF	p,Arg437G	10,07
chr7:116415099	G/A	G	MET	p,Ala1083T	10
chr4:55529137	G/A	G	KIT	p,?	10
chr8:128753236	G/A	G	MYC	p,?	9,97
chr1:11174433	G/A	G	MTOR	p,Val2414=	9,91
chrX:70339155	C/T	C	MED12	p,?	9,91
chr4:55104304	C/T	C	PDGFRA	p,?	9,9
chr8:38293118	C/T	C	FGFR1	p,?	9,87
chr12:25386023	C/T	C	KRAS	p,?	9,86
chr8:128752557	C/T	C	MYC	p,?	9,85
chr8:38283615	C/T	C	FGFR1	p,?	9,85
chr2:16084718	G/A	G	MYCN	p,?	9,84
chr2:29445247	C/T	C	ALK	p,Asp1160	9,84
chr7:140482803	G/A	G	BRAF	p,?	9,74
chr12:58145388	C/T	C	CDK4	p,Arg38Lys	9,67
chr2:29497994	G/A	G	ALK	p,Pro671Le	9,67
chr7:140481440	C/T	C	BRAF	p,Gln456=	9,66
chr7:116415177	C/T	C	MET	p,?	9,66
chr12:58145443	C/T	C	CDK4	p,Val20Me	9,62
chr8:128748939	C/T	C	MYC	p,?	9,6
chr1:65312363	G/A	G	JAK1	p,Tyr652=	9,59
chr5:112173329	G/A	G	APC	p,Ala680Th	9,59
chr11:69460978	C/T	C	CCND1	p,?	9,46
chr2:29445292	G/A	G	ALK	p,?	9,35
chr2:212488777	G/A	G	ERBB4	p,?	9,3
chr11:69460835	G/A	G	CCND1	p,?	9,27
chr3:41266062	C/T	C	CTNNB1	p,Ala20Val	9,21
chr12:58144408	C/T	C	CDK4	p,?	9,1
chr2:16085513	G/A	G	MYCN	p,?	9,05
chr4:55594236	C/T	C	KIT	p,Leu647P	9
chr4:55594220	C/T	C	KIT	p,Leu641=	8,98
chr3:178927923	G/A	G	PIK3CA	p,?	8,88
chr8:38308304	G/A	G	FGFR1	p,?	8,88
chr5:112175517	C/T	C	APC	p,Pro1409I	8,8
chr5:176517742	G/A	G	FGFR4	p,?	8,76
chr5:176523308	G/A	G	FGFR4	p,Trp655Te	8,73
chr4:55104350	C/T	C	PDGFRA	p,?	8,65
chr7:92272984	C/T	C	CDK6	p,?	8,63
chr12:58144586	G/A	G	CDK4	p,?	8,49
chr9:5073822	G/T	G	JAK2	p,?	8,47
chr12:58144610	G/A	G	CDK4	p,?	8,45
chr7:92412275	C/T	C	CDK6	p,?	8,33
chr8:38308270	C/T	C	FGFR1	p,?	8,32
chr12:25391239	G/A	G	KRAS	p,?	8,32
chr12:58145367	C/T	C	CDK4	p,Gly45Asp	8,3
chr7:116417443	G/A	G	MET	p,Gly1105(	8,26
chr4:1797286	G/A	G	FGFR3	p,?	8,26
chr19:4117546	G/A	G	MAP2K2	p,Leu58=	8,21

chr12:25398222	C/T	C	KRAS	p,Asp33As	8,21
chr4:55566195	C/T	C	KIT	p,?	8,2
chr12:25398232	C/T	C	KRAS	p,Val29=	8,17
chr7:92273260	C/T	C	CDK6	p,?	8,09
chr10:123276972	G/A	G	FGFR2	p,Ala315=	7,93
chrX:66874538	C/T	C	AR	p,?	7,88
chr8:38287124	C/T	C	FGFR1	p,?	7,86
chr19:17945983	C/T	C	JAK3	p,Lys652=	7,69
chr2:29497930	G/A	G	ALK	p,?	7,69
chr1:115252204	C/T	C	NRAS	p,Ala146Tl	7,69
chr15:66727501	G/A	G	MAP2K1	p,Glu73Lys	7,62
chr2:29445268	G/A	G	ALK	p,Pro1153	7,61
chr15:66774187	C/T	C	MAP2K1	p,Asn221=	7,6
chr17:37883211	C/T	C	ERBB2 ,,,(	p,Gly1038=	7,59
chr1:65310527	G/A	G	JAK1	p,Leu721P	7,53
chr4:55566243	C/T	C	KIT	p,?	7,32
chr12:25386945	G/A	G	KRAS	p,?	7,31
chr8:38283649	C/T	C	FGFR1	p,Asp277A	7,27
chr17:29422242	C/T	C	MIR4733 ,,	p,?	7,25
chr15:66774095	G/A	G	MAP2K1	p,Val191Ile	7,24
chr7:92292468	G/A	G	CDK6	p,?	7,23
chr4:55097750	C/T	C	PDGFRA	p,?	7,19
chr17:37881037	G/A	G	ERBB2	p,Cys789T	7,15
chr7:92286897	G/A	G	CDK6	p,?	7,12
chr8:38271760	G/A	G	FGFR1	p,Ser730P	7,09
chr4:1797300	G/A	G	FGFR3	p,?	7,08
chr1:11174447	G/A	G	MTOR	p,His2410T	7,01
chr7:55221916	G/A	G	EGFR	p,?	7
chr8:38271705	G/A	G	FGFR1	p,His748=	6,96
chr12:58144432	C/T	C	CDK4	p,?	6,93
chr11:69462006	G/A	G	CCND1	p,?	6,91
chr5:176519577	C/T	C	FGFR4	p,?	6,89
chr17:41256216	C/T	C	BRCA1	p,Val122Ile	6,88
chrX:66938978	C/T	C	AR	p,?	6,86
chr7:92286829	G/A	G	CDK6	p,?	6,83
chr7:55221800	G/A	G	EGFR	p,Glu282Ly	6,82
chr2:29436958	A/G	A	ALK	p,?	6,76
chr8:38282154	C/T	C	FGFR1	p,Gly301As	6,68
chr7:55221875	G/A	G	EGFR	p,?	6,67
chr3:178927994	G/A	G	PIK3CA	p,Trp424Te	6,64
chr8:38271738	C/T	C	FGFR1	p,Val737=	6,62
chr17:29422354	G/A	G	MIR4733 ,,	p,Trp9Ter	6,58
chr4:55104322	C/T	C	PDGFRA	p,?	6,58
chr17:41256249	C/T	C	BRCA1	p,Glu111Ly	6,56
chr10:43615598	T/C	T	RET	p,Phe893L	6,53
chr7:128849179	C/T	C	SMO	p,Cys469=	6,51
chr8:38308608	C/T	C	FGFR1	p,?	6,47
chr8:38271696	G/A	G	FGFR1	p,Asp751=	6,45
chr7:55221830	G/A	G	EGFR	p,Val292M	6,45
chr12:25398298	C/T	C	KRAS	p,Val7=	6,38
chr10:43609176	C/T	C	RET	p,?	6,38
chr8:38271691	G/A	G	FGFR1	p,Pro753Le	6,3
chr12:25364863	C/T	C	KRAS	p,?	6,22
chr17:37872384	C/T	C	ERBB2	p,?	6,21
chr7:128849205	C/T	C	SMO	p,Ala478Va	6,17
chr15:66774172	C/T	C	MAP2K1	p,Ile216=	6,17
chr12:25387258	G/T	G	KRAS	p,?	6,15
chr8:38271731	G/A	G	FGFR1	p,Leu740P	6,11
chr19:17948840	C/T	C	JAK3	p,Lys534=	6,1

chr8:38271501	G/A	G	FGFR1	p,Gln774T	6,07
chr19:3114977	G/A	G	GNA11	p,Gly171A	6,03
chr3:12645662	G/A	G	RAF1	p,Thr269=	6
chr7:55227963	G/A	G	EGFR	p,Trp477T	5,95
chr3:178927908	G/A	G	PIK3CA	p,?	5,94
chr12:25386073	C/T	C	KRAS	p,?	5,88
chr19:4117642	C/T	C	MAP2K2	p,?	5,88
chr3:178947852	C/T	C	PIK3CA	p,Phe909=	5,85
chr5:176524296	C/T	C	FGFR4	p,Tyr719=	5,8
chr2:29416657	C/T	C	ALK	p,Arg1432	5,8
chr5:176519584	C/T	C	FGFR4	p,?	5,73
chr19:3114987	C/T	C	GNA11	p,Pro174=	5,73
chr4:55097757	C/T	C	PDGFRA	p,?	5,67
chrX:70339164	C/T	C	MED12	p,?	5,66
chr12:58142329	C/T	C	CDK4 ,,,(3)	p,?, p,Lys2	5,66
chr5:176524276	C/T	C	FGFR4	p,?	5,64
chr2:16084302	C/T	C	MYCN	p,?	5,64
chr1:11189848	G/A	G	MTOR	p,Gly1887	5,51
chr7:92245651	G/A	G	CDK6	p,?	5,43
chr7:55248979	C/T	C	EGFR ,,,(2	p,?	5,41
chr8:38271551	G/A	G	FGFR1	p,?	5,4
chr7:55227981	C/T	C	EGFR	p,Thr483Il	5,38
chr8:38282194	G/A	G	FGFR1	p,Leu288=	5,35
chr2:29416599	C/T	C	ALK	p,Ala1452	5,35
chr11:69466648	T/C	T	CCND1	p,?	5,26
chr3:178927928	G/A	G	PIK3CA	p,?	5,26
chr7:128845511	G/A	G	SMO	p,Val270Il	5,26
chr12:56478881	G/A	G	ERBB3	p,Gly113A	5,23
chr17:37882885	G/A	G	ERBB2 ,,,(	p,Arg981=	5,21
chr19:17946019	G/A	G	JAK3	p,Asp640=	5,19
chr19:17946013	G/A	G	JAK3	p,Gly642=	5,18
chr12:58144631	G/A	G	CDK4	p,?	5,18
chr7:128845606	G/A	G	SMO	p,Met301Il	5,18
chr8:38308672	C/T	C	FGFR1	p,?	5,17
chr1:115256538	G/A	G	NRAS	p,Thr58Ile	5,15
chr4:1797216	G/A	G	FGFR3	p,?	5,15
chr2:212488743	G/A	G	ERBB4	p,Gly702=	5,15
chr2:29416566	C/T	C	ALK	p,Val1463I	5,15
chr19:3115029	C/T	C	GNA11	p,Gly188=	5,14
chr10:43615633	C/T	C	RET	p,Ser904=	5,13
chr17:37882912	G/A	G	ERBB2 ,,,(	p,Gln990=	5,11
chr5:176523315	C/T	C	FGFR4	p,Pro658S	5,1
chr17:37879872	G/A	G	ERBB2	p,Val723M	5,1
chr17:41215988	C/T	C	BRCA1	p,?	5,03
chr7:116339342	C/T	C	MET	p,Asn68=	5,03
chr11:102238604	C/T	C	BIRC2	p,?	5,02
chr7:55198974	C/T	C	EGFR	p,?	5
chr8:38308646	C/T	C	FGFR1	p,?	4,99
chr2:16084298	C/T	C	MYCN	p,?	4,99
chr12:56478868	C/T	C	ERBB3	p,Thr108=	4,98
chr12:58142896	C/T	C	CDK4 ,,,(3)	p,?, p,?	4,96
chr7:55249100	G/A	G	EGFR ,,,(2	p,Asp800A	4,96
chr7:128845529	G/A	G	SMO	p,Val276M	4,94
chr8:38271495	G/A	G	FGFR1	p,Pro776S	4,93
chr7:140434416	C/T	C	BRAF	p,Gly761A	4,88
chr8:38315027	G/A	G	FGFR1	p,Leu13Ph	4,88
chr12:56478883	G/A	G	ERBB3	p,Gly113=	4,86
chr4:55589744	C/T	C	KIT	p,?	4,85
chr7:116340217	C/T	C	MET	p,Ser360P	4,84

chr7:128845555	G/A	G	SMO	p,Gln284=	4,82
chr7:116423482	G/A	G	MET	p,Glu1271l	4,8
chr7:140507859	C/T	C	BRAF	p,Glu204=	4,76
chr3:178928107	C/T	C	PIK3CA	p,Thr462Ile	4,75
chr12:25398187	G/A	G	KRAS	p,?	4,74
chr1:11184640	G/A	G	MTOR	p,Arg2193	4,73
chr19:17945970	G/A	G	JAK3	p,Arg657T	4,73
chr2:29436969	C/T	C	ALK	p,?	4,71
chr15:66729138	C/T	C	MAP2K1	p,Gln116T	4,7
chr2:212488718	G/A	G	ERBB4	p,Arg711C	4,7
chr4:55594259	G/A	G	KIT	p,Val654=	4,66
chr4:55097730	G/A	G	PDGFRA	p,?	4,64
chr4:55097832	C/T	C	PDGFRA	p,?	4,64
chr11:69462023	G/A	G	CCND1	p,?	4,63
chr11:533870	C/T	C	HRAS	p,Glu62=	4,62
chr4:55097741	C/T	C	PDGFRA	p,?	4,62
chr12:58144953	C/T	C	CDK4	p,?	4,61
chr2:16083355	G/A	G	MYCN	p,?	4,6
chr7:140507798	C/T	C	BRAF	p,Glu225Ly	4,56
chr5:176519602	G/A	G	FGFR4	p,?	4,55
chr8:128748916	T/C	T	MYC	p,?	4,54
chr10:43609146	C/T	C	RET	p,?	4,52
chr2:16080700	G/A	G	MYCN ,,,(2	p,?	4,51
chr4:55097812	C/T	C	PDGFRA	p,?	4,5
chr11:102234251	G/A	G	BIRC2	p,?	4,48
chr11:102238627	C/T	C	BIRC2	p,?	4,47
chr2:16086566	C/T	C	MYCN	p,?	4,44
chr12:58144971	G/A	G	CDK4	p,?	4,41
chr5:176517416	G/A	G	FGFR4	p,Glu39=	4,41
chr6:152420028	G/A	G	ESR1	p,Gly572As	4,41
chr17:37881430	G/A	G	ERBB2	p,Glu874=	4,4
chr8:38308633	C/T	C	FGFR1	p,?	4,4
chr19:3118897	G/A	G	GNA11	p,?	4,32
chr4:55152099	T/C	T	PDGFRA	p,Met844T	4,26
chr5:176519572	G/A	G	FGFR4	p,?	4,25
chr10:43609959	G/A	G	RET	p,Val637=	4,23
chr17:37881000	G/A	G	ERBB2	p,Val777M	3,98
chr7:55228009	C/T	C	EGFR	p,Ser492=	3,92
chr7:55233043	G/A	G	EGFR	p,Gly598G	3,75
chr7:55249071	C/T	C	EGFR ,,,(2	p,Thr790M	3,55
chr10:43609953	C/C	C	RET		3,51
chr17:37879658	G/A	G	ERBB2	p,Arg678G	2,55
chr11:534289	C/T	C	HRAS	p,Gly12Ser	2,45
chr4:55593621	A/G	A	KIT	p,Ile563Va	1,88
chr11:534285	C/C	C	HRAS		1,3
chrX:66943543	C/C	C	AR		1,2
chr7:128845571	C/C	C	SMO		1,1
chr12:56482537	G/G	G	ERBB3		1,05
chr17:37868207	T/T	T	ERBB2		0,85
chr10:43617397	C/C	C	RET		0,62
chr19:17949164	T/T	T	JAK3		0,56
chr4:1807889	A/A	A	FGFR3		0,55
chr4:1803565	G/G	G	FGFR3		0,55
chr12:25380276	T/T	T	KRAS		0,45
chr7:55211079	A/A	A	EGFR		0,42
chr4:55141036	T/T	T	PDGFRA		0,39
chr15:90631839	T/T	T	IDH2		0,38
chr3:178916945	A/A	A	PIK3CA		0,38
chr4:55599321	A/A	A	KIT		0,37

chr3:41266136	T/T	T	CTNNB1	0,32
chr7:140453133	T/T	T	BRAF	0,31
chr4:55593610	T/T	T	KIT	0,3
chr3:178936073	C/C	C	PIK3CA	0,3
chr3:178936071	A/A	A	PIK3CA	0,3
chr3:178936093	G/G	G	PIK3CA	0,3
